# Bibliometric and Visualization Analysis of Path Planning and Trajectory Tracking Research for Autonomous Vehicles from 2000 to 2025

**DOI:** 10.3390/s26030964

**Published:** 2026-02-02

**Authors:** Bo Niu, Roman Y. Dobretsov

**Affiliations:** Institute of Machinery, Materials, and Transport, Peter the Great St. Petersburg Polytechnic University, 195251 St. Petersburg, Russia; nyu2.b@edu.spbstu.ru

**Keywords:** path planning, trajectory tracking, autonomous vehicles, bibliometric analysis, visualization analysis

## Abstract

**Highlights:**

**What are the main findings?**

- A systematic bibliometric and visualization analysis fills the gap in quantitative reviews of autonomous vehicle path planning and trajectory tracking.
- Research hotspots and frontier topics are identified, revealing the evolution from traditional control to optimization and learning-based methods.

**What are the implications of the main findings?**

- By moving beyond descriptive literature analysis toward deeper technical insight, this study clarifies the internal logic and development pathways of the field.
- The revealed trends and emerging directions provide explicit guidance for future research on intelligent planning, decision-making, and control of autonomous vehicles.

**Abstract:**

With the rapid development of the automotive industry, autonomous driving has attracted growing research interest, among which path planning and trajectory tracking play a central role. To better understand the evolution, current status, and future directions of this field, this study conducts a comprehensive bibliometric analysis combined with latent Dirichlet allocation (LDA) topic modeling on publications related to autonomous vehicle path planning and trajectory tracking indexed in the Web of Science database. Multiple dimensions are examined, including publication trends, highly cited authors, leading institutions, research domains, and keyword co-occurrence patterns. The results reveal a sustained growth in research output, with trajectory planning, path optimization, trajectory tracking, and model predictive control (MPC) emerging as dominant topics, alongside a notable rise in learning-based approaches. In particular, reinforcement learning (RL) and deep reinforcement learning (DRL) have become increasingly prominent in complex decision-making and tracking control scenarios. The analysis further identifies core contributors and institutions, highlighting the leading roles of China and the United States in this research area. Overall, the findings provide a systematic overview of the knowledge structure and evolving research trends, offering valuable insights into key opportunities and challenges and supporting future research toward safer and more intelligent autonomous driving systems.

## 1. Introduction

With the continuous rise in the global fleet of various vehicles and the rapid advancement of multidisciplinary technologies, the application scenarios for vehicles are becoming increasingly diverse. Against this backdrop, autonomous driving technology has emerged. This technology can effectively reduce traffic accidents caused by driver error, significantly enhancing road safety. Simultaneously, it can make a significant impact in the logistics and transportation sector, reducing reliance on human labor and improving transport efficiency. Furthermore, autonomous driving technology can be widely applied to public transportation systems, optimizing traffic flow, alleviating congestion, and bringing comprehensive transformation and improvement to modern transportation. Within the autonomous driving technology framework, the vehicle planning and control segment serves as the core link of the autonomous driving system, playing a pivotal bridging role. It not only acts as the primary driver for enhancing the intelligence level of autonomous vehicles (AVs) but also provides crucial support for ensuring driving safety. Therefore, conducting thorough investigations into path planning and trajectory tracking technologies is essential for advancing the development of AVs [1].

Unlike traditional driving, autonomous driving technology enables real-time decision-making and route planning, rapidly calculates optimal paths, and employs advanced algorithms to ensure vehicles smoothly and precisely follow target trajectories. Path planning is a multidisciplinary technique that amalgamates advanced domains including computer science, machine learning, and robotics. It aims to enable AVs, robots, drones, and other mobile entities to precisely and efficiently plan optimal or feasible routes from origin to destination within complex environments. It not only considers the shortest path but also balances key factors such as energy consumption minimization, obstacle avoidance, and time constraints, ensuring the comprehensiveness, practicality, and efficiency of path planning [2].

In autonomous driving, path planning first generates a global route through a road network; subsequently, local planners handle moment-to-moment maneuvers to avoid obstacles. Among these, global route—also known as navigation planning—has extensive applications in robot navigation and map navigation domains, with related algorithms having reached a relatively mature stage. In 1956, Dutch computer scientist Dijkstra introduced an algorithm that bears his name. It starts from the source vertex and greedily scans every node in the graph to construct the minimum-cost route [3]. Building upon this foundation, scholars such as Hart and Nilsson introduced heuristic functions and proposed the A* algorithm. By prioritizing the expansion of nodes with strong goal orientation, this approach significantly reduces the number of nodes searched and enhances search efficiency [4]. Meng and Wang integrate the global-level semantic priors encapsulated in large language models with the exact heuristic search of the A* algorithm, yielding a hybrid planner that prunes the search space and reduces both time complexity and memory footprint by an order of magnitude. This approach enhances algorithmic efficiency while maintaining path validity, making it suitable for path planning in large-scale scenarios [5]. The RL-RRT algorithm proposed by Chiang et al. employs DRL to learn obstacle avoidance strategies, utilizing it as a local optimizer. Subsequently, a reachability-based distance heuristic is employed to steer the tree extension, efficiently resolving path-planning tasks subject to intricate constraints [6].

In practical path planning tasks, the most challenging aspect is often local path planning, also known as trajectory planning. Consequently, the field of trajectory planning has attracted significant academic attention, with research efforts relatively concentrated in this area. Xu et al. propose a motion planning method that accounts for uncertainty, enabling AVs to generate safe trajectories in complex traffic environments. This method generates candidate trajectories through spatiotemporal grid points, combines a Linear Quadratic Gaussian (LQG) framework with a Kalman filter [7], and optimizes trajectory safety. Its effectiveness has been validated through experiments. Nguyen et al. proposed a hierarchical motion planning and offline robust MPC strategy for AVs in complex scenarios [8]. Through the artificial potential field method and offline Robust MPC (RMPC) method, this strategy effectively addresses challenges under adverse conditions, ensuring safe and stable vehicle operation. Ding et al. introduced a trajectory planning methodology grounded in spatiotemporal semantic corridors, which integrates semantic information with spatiotemporal constraints to effectively enhance the safety of AVs in complex urban environments [9]. Zhejiang University and Cainiao Unmanned Vehicle Technology Team proposed an autonomous driving trajectory planning system called CarPlanner, which is an autoregressive planner based on RL seeks to address the trajectory planning challenge in extensive autonomous driving systems [10]. We demonstrate its effectiveness on the real-world dataset nuPlan and show its ability to surpass existing State of the Art (SOTA) approaches combining rules and imitation learning. Recent research by Gao Fei’s team at Zhejiang University has proposed an innovative hierarchical trajectory planning method. This method mimics the intuitive human ability in path planning, comprehensively considering global environmental data and historical experience to determine viable routes. This approach enhances both computational robustness and motion fidelity through a spatiotemporal trajectory optimizer designed for numerical stability, alongside a novel two-layer polynomial trajectory structure. Leveraging differential flatness, the optimizer boosts efficiency while inherently resolving singularities in the original formulation, ensuring reliable convergence to smooth, feasible trajectories during complex maneuvers [11].

Trajectory tracking technology is the key to enabling AVs to travel along a predetermined path, and to correct deviations in real time and adjust direction and speed through a feedback control system [12]. The Pure Pursuit Algorithm and Stanley algorithm are control strategies designed based on vehicle geometry. They possess a straightforward structure and are facile to deploy, rendering them appropriate for fundamental driving scenarios. To secure pinpoint tracking under demanding, high-speed conditions, designers often turn to optimization-based strategies like Linear Quadratic Regulator (LQR) and MPC. Lu et al. proposed an adaptive LQR controller based on genetic algorithms. They generated an offline parameter table by optimizing the weight matrix and developed an online adaptive controller. The simulation results showed that it enhances trajectory fidelity and stabilizes the vehicle across the full speed spectrum [13]. Zhao et al. proposed a trajectory tracking controller based on linear active disturbance rejection control, which improves the tracking accuracy and dynamic performance of ground AVs in complex environments through modeling compensation and extended disturbance observation [14]. A research team from Anhui University of Technology proposed a curvature-adaptive Linear Time-Varying MPC (LTV-MPC) control algorithm [15] to address the issues of diminished tracking precision and inadequate driving stability of vehicles on trajectories with changing curvature (such as curves and ramps). To mitigate the recurring trajectory-tracking issue encountered by AVs in specific working scenarios, especially in the presence of unknown iterative change interference and input constraints, Zhang et al. proposed an enhanced adaptive iterative learning control scheme rooted in the internal model principle. The integration of Adaptive High-Order Internal Model (AHOIM) enhances static precision and dynamic response while the system retraces the same path [16]. To address extreme working conditions such as tire blowouts, Yang et al. advanced a trajectory-tracking strategy that hinges on the Frenet coordinate system. The Pontryagin Maximum Principle (PMP) was used to optimize speed control in the longitudinal direction, and back-stepping was used in the lateral direction to ensure Lyapunov stability [17]. This method showed good control effects in both simulations and actual vehicle experiments.

Currently, the field of planning and control technology is developing rapidly. However, there is still a lack of research that comprehensively sorts out and systematically summarizes its progress in academic literature at home and abroad. Traditional literature review methods face problems of low efficiency and poor timeliness when processing large amounts of literature, which makes it difficult to meet the needs of quickly and accurately obtaining key information. Therefore, an efficient literature induction and summary method is needed to systematically analyze the development of this field. Bibliometric analysis is an interdisciplinary quantitative research method that integrates mathematics, statistics, and bibliographic theory. It mainly conducts quantitative research on academic literature and its related elements. The analysis encompasses the quantity of publications, including journal papers and citation data, author groups (individual authors and research institutions), and document identifiers (such as subject terms, keywords, etc.) [18]. This method reveals the present condition and developmental trajectories of research within a particular domain via quantitative analysis, forms objective conclusions, and uses visualization technology to transform complex data into intuitive maps to help identify disciplinary hotspots and cutting-edge directions. By coupling CiteSpace(version 6.2.4, Drexel University, Philadelphia, PA, USA) or VOSviewer (version 1.6.19, Centre for Science and Technology Studies, Leiden University, Leiden, Netherlands) (for citation, co-word, and cluster analysis) with Statistical Package for the Social Sciences, researchers can drill down into the intellectual core of a field [19]. Although both domestic and international researchers have undertaken comprehensive research on autonomous vehicle path planning and trajectory tracking technology and produced many review results, there is still a lack of research that systematically reviews the development trends in this field based on bibliometric methods. Therefore, it is necessary to carry out an in-depth analysis using bibliometric approaches.

To clarify the basis of our classification, the representative review studies summarized in Table 1 were identified through a review-focused screening procedure (document type “Review”), complemented by backward and forward citation tracking. The comparison is organized along five reporting dimensions—Scope, Evidence base, Coverage window, Methodology, and Outputs—which together describe (1) what part of the planning–control pipeline a review covers, (2) how its evidence is constructed, and (3) what types of conclusions it can support. This multi-dimensional scheme is used to position the present work relative to existing review paradigms and to reduce redundancy. Compared with traditional narrative reviews, research methods based on visualized bibliometric analysis have demonstrated more prominent advantages in practice, as summarized in Table 1.

To enhance the completeness of review identification and to minimize redundancy with existing surveys, we additionally conducted a review-focused screening and citation tracking of representative reviews. The detailed retrieval strategy and screening workflow are described in Section 2.3. Based on this validated positioning, compared with traditional reviews, the present study has the following advantages and distinctive features:

(1) Most existing review studies in this field focus on a specific module of the autonomous driving planning–control pipeline or on predefined application scenarios. In contrast, this study is organized around the end-to-end planning–control chain, without being restricted to a single method or a single scenario. Using a dynamic time-window design for systematic analysis, it covers mainstream research across multiple stages and the evolution of different technical paradigms, thereby constructing a higher-level knowledge map of the field.

(2) Many existing studies rely on author-selected literature, which may suffer from incomplete coverage and selection bias and is often difficult to reproduce. In this study, 329 publications were retrieved and screened through a systematic search of the Web of Science Core Collection (WoSCC), providing broader coverage, reduced bias, and stronger reproducibility; therefore, the resulting trend and frontier analyses are more convincing.

(3) Methodologically, most prior work is dominated by narrative reviews or inductive summaries from a limited perspective. By contrast, this study employs multiple complementary methods for cross-validation, enabling a more objective characterization of the knowledge structure and a clearer identification of research hotspots and the evolution of emerging frontiers, thereby yielding more systematic conclusions.

(4) In terms of outputs, existing reviews mainly emphasize content categorization and problem summarization, whereas this study further provides results on research trends, leading authors/institutions, hotspots/frontiers, and thematic evolution. These findings can more directly support topic positioning and research roadmap planning.

(5) Traditional reviews are prone to researchers’ subjective preferences, and important studies may be inadvertently overlooked. In this study, bibliometric tools such as CiteSpace apply threshold-based filtering to the literature data and suppress long-tail and redundant linkage noise, thereby preserving the backbone structure of the research field.

Therefore, studies adopting this bibliometric approach are of significant value for comprehensively capturing and monitoring the field’s development trajectory.

Accordingly, this study adopts bibliometric methods and conducts a systematic analysis through the following steps: first, export indexed articles from WoSCC for downstream analysis, then use quantitative analysis methods for statistical processing, identify influential authors, countries and research institutions in the field utilizing citation analysis, and subsequently integrating keyword co-occurrence analysis to reveal the evolution process and research hotspots of the field [24]. The remainder of this article is structured in four sections: research design and data sources are outlined in Section 2; Section 3 maps the current landscape and hotspots of autonomous-vehicle path planning and trajectory tracking; Section 4 pinpoints emerging trends; and Section 5 translates these insights into a forward-looking research agenda.

## 2. Research Methods and Materials

### 2.1. Research Methods

#### 2.1.1. Bibliometric Analysis Method

Bibliometrics is a visualization technology that conducts comprehensive and detailed statistical analysis and scientific and precise quantitative analysis of massive amounts of published literature. It can clearly present the overall status, development context, and future dynamic trends of a specific knowledge field in an intuitive and easy-to-understand visualization form. It is an important branch of information visualization [25]. In the field of academic research, Analytical and visualization instruments for literature, exemplified by CiteSpace, VOSviewer, and Pajek (version 5.18, University of Ljubljana, Ljubljana, Slovenia), are widely used, providing researchers with powerful data processing and visualization support. Among them, CiteSpace, developed by Professor Chen Chaomei’s team, is comprehensive in functions and is among the most extensively utilized tools for literary analysis and visualization in academia [19,26].

Based on visualization tools such as CiteSpace and VOSviewer, this paper systematically sorted out the core literature in the domain of autonomous vehicle path planning and trajectory tracking throughout the last two decades. A knowledge graph in this domain has been developed and studied [19]. The study focused on the dynamics of publication volume, author collaboration networks, the distribution of research institutions, keyword co-occurrence, emergent word analysis, and topic cluster evolution, comprehensively revealing the research evolution path and hotspot evolution trends in this field [27]. For details on the mapping method, please refer to the reference [28].

#### 2.1.2. Method for Analyzing Published Authors

In scientometrics, a core group of authors denotes a collective of writers who have produced a substantial volume of publications in a certain research landscape and have significant academic influence [29]. This group usually represents the research backbone and academic authority in the relevant landscape and has a significant position in guiding research directions and disseminating knowledge [30]. Therefore, identifying core authors is of great significance for gaining an in-depth understanding of the discipline development pattern and building a scientific research cooperation network.

According to Price’s Law, proposed by bibliometrician Derek J. de Solla Price, the identification of core authors can be estimated using the following empirical formula [31]:

(1) $$\;M_{P}=0.747\times \sqrt{N_{pmax}}$$

Among them, $M_{P}$ represents the publication number threshold of core authors, that is, the publication threshold for qualifying as a core author; $N_{Pmax}$ reflects the total publications credited to the author with the most publications in the research field; The constant 0.747 is an empirical coefficient obtained through empirical research.

The meaning of this formula is that if there is an author in a certain field whose publication volume is $N_{Pmax}$, consequently, an author who has written a minimum of $M_{P}$ publications may be regarded as a core author.

#### 2.1.3. Keyword Analysis Method

The keyword analysis in this paper uses VOSviewer software (version 1.6.19) [30] to perform knowledge graph clustering and Pajek software (version 5.18) [31] to perform layout optimization. To visually display the time characteristics of a keyword’s appearance, this article uses different colors to represent the average year a keyword appears. The mean publication year T of a keyword is computed as follows:

(2) $$\;T=\frac{\sum \left(year\cdot C_{year}\right)}{\sum C_{year}}$$

Among them, *year* refers to when the keyword first appears in print, and $C_{year}$ is the frequency of the keyword’s appearing in that year.

#### 2.1.4. Keyword Emergence Analysis Method

“Emergence” refers to the phenomenon that the frequency of a specific keyword increases significantly within a specified timeframe, which often marks the emergence of emerging areas of intensive investigation or cutting-edge directions [32]. By analyzing the temporal distribution of keywords, emergent word detection can identify keywords with a high frequency change rate and a fast growth rate, thereby revealing the evolution trend and development trend of research topics in a certain discipline. At the same time, in research, we can combine keyword co-occurrence networks with emergent word detection to elucidate the research landscape and developmental context in the domain of “autonomous driving vehicle path planning and trajectory tracking” in a more comprehensive and profound way.

In this study, we used the CiteSpace tool (version 6.2.4) [19] to detect emergent words in relevant literature within the domain of autonomous vehicle path planning and trajectory tracking. The core algorithm of this tool is based on the state machine model proposed by Kleinberg [33]. It can effectively identify the research focus and frontier shifts in this field at different times and provide an empirical basis for grasping the development of the discipline.

### 2.2. Autonomous Vehicle Algorithm System Architecture

**Figure 1:**
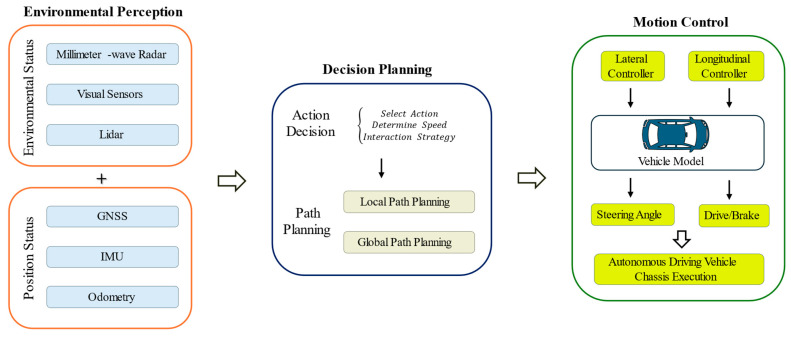
Autonomous vehicle algorithm system architecture.

The algorithm system architecture of an autonomous vehicle is a complex and highly integrated multi-layer architecture, which is usually composed of three core subsystems [34]: environment perception (Perception), decision planning (Planning), and motion control (Control). As shown in Figure 1, the typical architecture of the algorithm system of an autonomous vehicle is shown. The figure clearly shows the functions of each subsystem and its information interaction path, among which the planning and control parts play a core role in the autonomous driving vehicle algorithm system.

In an autonomous driving system, the planning part plays a vital role, and its primary function is to make behavioral judgments based on perceived environmental information. First, in the cognitive understanding stage, the system accurately identifies and judges the vehicle’s own position and surrounding environment by fusing multi-source sensor information. Then, in the decision planning stage, the system predicts future traffic conditions and formulates a secure and effective driving route from the present location to the destination [35]. This process involves complex algorithms (including but not limited to path optimization, collision avoidance, and dynamic traffic adaptation) to ensure the rationality and foresight of vehicle behavior [36,37]. The control part focuses on converting the decisions made in the planning stage into specific vehicle dynamic control instructions. Deeply integrated with the vehicle’s underlying control systems, the control layer utilizes advanced drive-by-wire technology to precisely control actuators such as electronic braking, drive, and steering. This process not only requires real-time monitoring and adjustment of vehicle responses based on an accurate vehicle model [38] to ensure high consistency between the driving trajectory and the planned path [39], but also requires consideration of vehicle stability and safety to achieve precise management of vehicle dynamic behavior. Overall, the control layer is the key link in the autonomous driving system that bridges the gap between vehicle behavior decision-making and physical execution.

### 2.3. Origins of Data and Retrieval Methods

The literature dataset was retrieved from the WoSCC, which is widely used in bibliometric studies due to its standardized bibliographic metadata and citation indexing. Using the advanced search function, we systematically collected publications related to autonomous-vehicle path planning and trajectory tracking within the predefined time span of 2000–2025 (with 2025 records included up to September). The search was conducted using topic-term combinations (Table 2) that covered core phrases such as “autonomous vehicle”, “self-driving/driverless”, “path planning”, and “trajectory tracking”, together with their synonyms, connected by Boolean operators (AND/OR) [40]. The document types were restricted to English-language articles and reviews.

**Figure 2:**
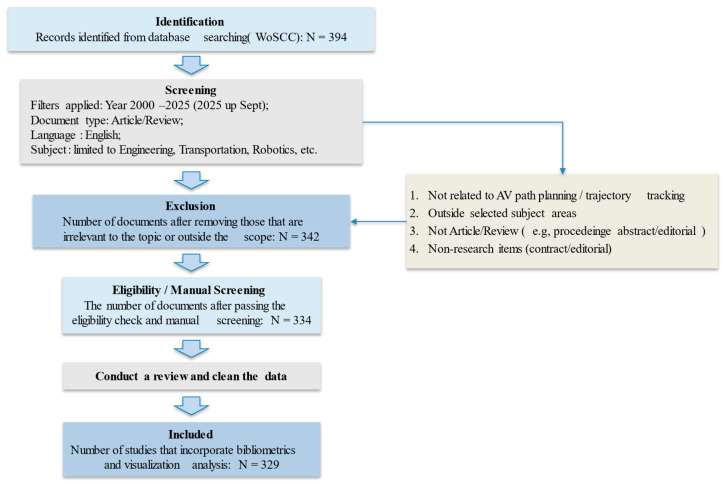
PRISMA literature screening flowchart.

To improve topical precision, we optimized the screening procedure following standard systematic-review and bibliometric practices [41]. We first applied a discipline/research-area filter (e.g., engineering, transportation, and robotics-related areas) to remove evidently irrelevant records. Next, we manually assessed titles, abstracts, and author keywords against predefined inclusion and exclusion criteria. After removing duplicates and cleaning the metadata, we retained 329 eligible publications with complete bibliographic information (title, authors, affiliations, keywords, abstract, references, and citation data). The overall screening workflow is shown in Figure 2.

To ensure comprehensive coverage of potentially related review studies, we additionally conducted a review-focused retrieval and cross-check procedure. Specifically, we screened records indexed as the document type “Review” and performed complementary searches using review-indicative terms (e.g., “review”, “survey”, “bibliometric”, and “mapping”) combined with the domain keywords. We also performed backward and forward citation tracking for the representative reviews summarized in Table 1 (via reference lists and citing-article links) to identify additional relevant review studies that may not be captured by keyword-based retrieval alone. All identified review records were assessed using the same inclusion/exclusion criteria and incorporated into the comparative discussion where appropriate.

To reduce potential subjectivity in the eligibility assessment and manual screening, we introduced supplementary quantitative rules based on bibliographic metadata. Specifically, we performed a rule-based relevance check using the title, abstract, and author keywords of each record. Only studies that simultaneously satisfied the following criteria were retained: (1) the record contains at least one entity term related to AVs (e.g., autonomous driving/self-driving/driverless vehicles); and (2) the record contains at least one term related to path planning and/or trajectory tracking (e.g., path/trajectory/motion planning, trajectory tracking/path following).

## 3. Analysis of Basic Characteristics of Autonomous Vehicle Path Planning and Trajectory Tracking Research

### 3.1. Descriptive Statistics

**Figure 3:**
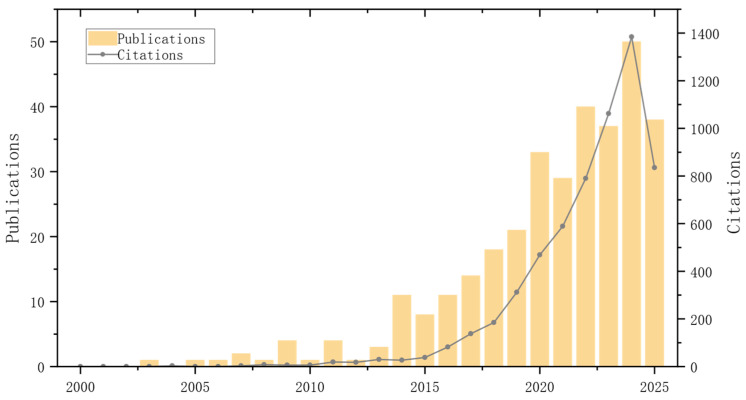
Number of scientific publications and citations for autonomous vehicle path planning and trajectory tracking research from 2000 to 2025.

Temporal changes in publication output can elucidate the evolution and developmental trajectories of an academic domain [32], and this is a classic scientometric approach for tracking disciplinary development. Annual publication counts are a key indicator of a research direction’s development and future trends. Trends in the cumulative number of publications can further distinguish development stages (e.g., formation, growth, and explosive stages) [42]. Figure 3 presents the research output over nearly two decades. From 2000 to 2013, the field experienced a slow start-up phase with relatively few publications, suggesting it was still at an early stage. Since 2014, publications have shown steady growth, indicating increasingly active research and gradually rising academic influence. After 2020, this growth became more pronounced, and the average growth rate accelerated, consistent with the broader expansion of artificial intelligence-related fields [43]. Publication output peaked at about 50 in 2024. Although there was some decline between 2021 and 2023, the overall level remained high. Moreover, based on the current annual progress, trend analysis, and the scientific growth model [44], publications in 2025 are expected to surpass the historical high and reach a new peak.

Between 2000 and 2010, the number of citations in this research field remained relatively low, with little change. This indicates the comparatively restricted impact and acknowledgement of research in this domain during that period, which is consistent with the typical characteristics of the early stages of knowledge diffusion in emerging fields [43]. However, since 2011, the number of citations has begun to show a gradual and weak upward trend, indicating that the research results are gradually receiving more attention. After entering 2015, the growth rate of citations has accelerated significantly, indicating that the influence and recognition of research results have increased significantly, indicating that the field may have entered a stage of exponential growth [45]. Especially since 2018, the growth rate of citations has increased significantly, and this trend has reached explosive growth between 2021 and 2024, which is particularly significant. Although the citation data for 2025 (based on existing forecasts) is still at a relatively low level (mainly due to the inherent time lag of citation behavior), an examination predicated on the dynamic model of citation proliferation [46] and the long-term citation growth trend in the field suggests that the number of citations in 2025 is likely to reach a new historical peak.

The quantitative analysis in Figure 3 shows that after 2011, research output in the domain of autonomous vehicle path planning and trajectory tracking has shown a significant leap: the quantity of publications and the rate of citations have increased exponentially [45], indicating that this research direction is undergoing a period of rapid knowledge accumulation, and its academic attention and the influence of its results continue to increase. This phenomenon is consistent with the characteristics of the definition of emerging research frontiers in scientometrics [47], and strongly reflects the systematic attention and in-depth exploration of this field by the academic community.

### 3.2. Analysis of Lead Authors

Based on statistical data, Table 3 lists the authors possessing the greatest quantity of publications within the domain of autonomous vehicle path planning and trajectory tracking, along with their personal information (ranking is not considered if the number of publications is the same; for authors with only three publications, only a subset is selected). Statistical results show that the most prolific author contributed five papers. According to the core author calculation formula proposed in Price’s Law $N_{Pmax}=5$, the threshold $M_{P}\approx 1.67$ papers are obtained, which is rounded to 2 papers. That is, the author who has published two papers is defined as the core author in the field. Based on this criterion, a total of 94 authors meet the definition of core authors. The identification of core author groups helps to define the group of researchers who have made major contributions to the production of knowledge within the domain, and whose scientific findings have significantly advanced the field.

**Figure 4:**
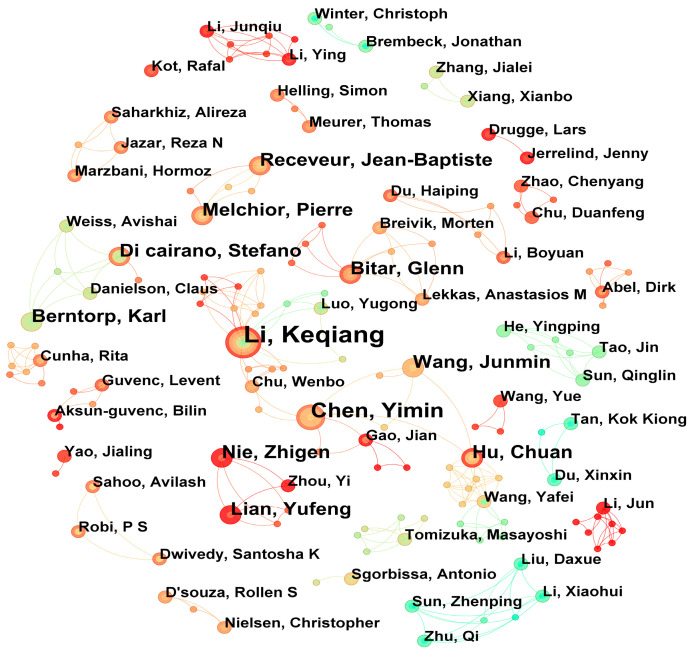
Author collaboration network.

As shown in Figure 4 and Table 3 of this study, Li Keqiang (5 papers) and Chen Yimin (4 papers) are the most important nodes in the network, both from China, which is consistent with the phenomenon observed in other studies that highly productive core authors often dominate the collaboration network [48]. The nodes of authors who published 3 articles are dense, which also reflects the widespread existence of “weak connection” advantages in the collaborative network. By drawing on the optimization ideas of path-finding algorithms, we analyzed the author collaboration network in this field and identified four main collaboration teams (Li Keqiang team, Chen Yimin team, Bitar Glenn team, and Nie Zhige team). To some extent, these teams are similar to key nodes or hubs in a path-finding network, significantly influencing the dissemination of knowledge and the efficacy of collaboration [49]. In the future, it may be possible to further explore the application of these algorithm optimization principles in predicting or enhancing the potential of scientific research cooperation.

In the process of conducting an in-depth analysis of academic achievements in related fields, we focused on authors who published two or more papers. The authors with the highest citation counts are presented in Table 4. Sahoo A., Dwivedy S. K., and Robi P. S. co-authored the two most-cited articles and tied for first place, which clearly demonstrates their strong influence and academic standing in the field. In addition, Li Keqiang ranked first in publication count and second in citation frequency, reflecting both his solid research capability and academic impact. His work is not only substantial in quantity but also high in quality, and has been widely recognized and cited. Meanwhile, Chen Yimin (four publications) and several other scholars with three publications produced a considerable number of papers, but their citation impact remained below the global average. Due to relatively low citation counts, they did not enter the ranking shown in Table 4. This also suggests that academic influence is determined not only by publication volume, but that citation frequency is another key indicator of academic value and impact.

### 3.3. Publishing Institutions and Country

This study analyzes research institutions that have published two or more papers and examines their academic influence in autonomous vehicle path planning and trajectory tracking. By systematically sorting and analyzing relevant data, the top ten institutions by citation frequency were identified, as shown in Table 5. Among them, Tsinghua University, the Norwegian University of Science and Technology (NTNU), and Beijing Institute of Technology have the highest citation impact. These three institutions have produced influential results in autonomous driving research, attracting extensive attention and citations and making substantial contributions to the advancement of the field. Although NTNU published only six papers, they have been highly cited, indicating high-quality research outcomes with strong influence and broad recognition in the academic community. Further analysis shows that five of the top ten most-cited institutions are from China, suggesting that China has made substantial progress in autonomous driving technology research and demonstrates strong research capacity in this domain [50]. The achievements of Chinese institutions have supported domestic technological development and contributed to global advances in autonomous driving, highlighting China’s research strength and influence in this area.

**Figure 5:**
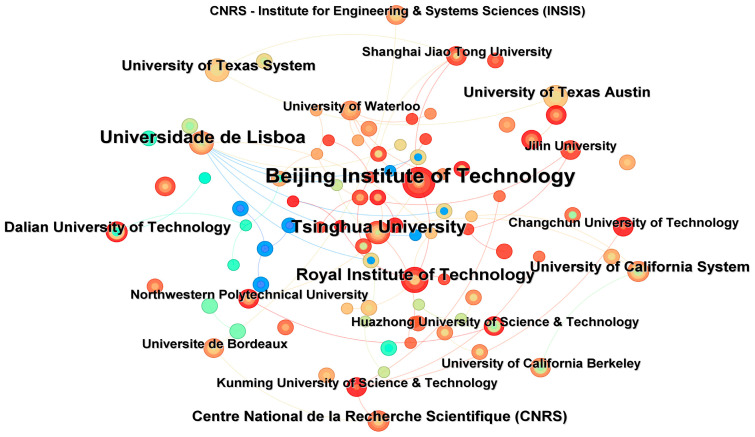
Visualization of the publishing institutions (number of publications).

**Figure 6:**
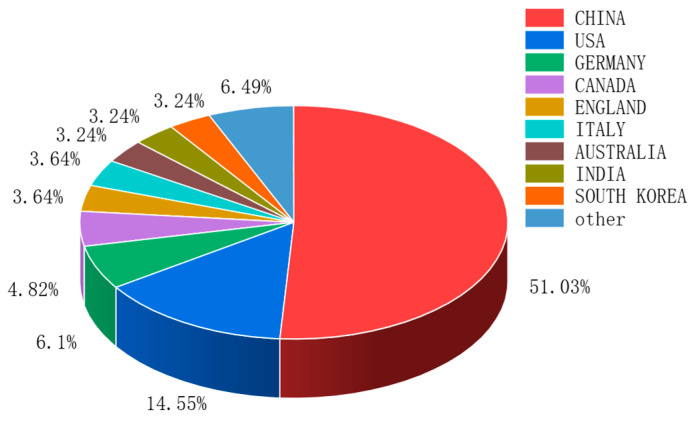
Percentage of publications by country (countries with 2 or more publications).

**Figure 7:**
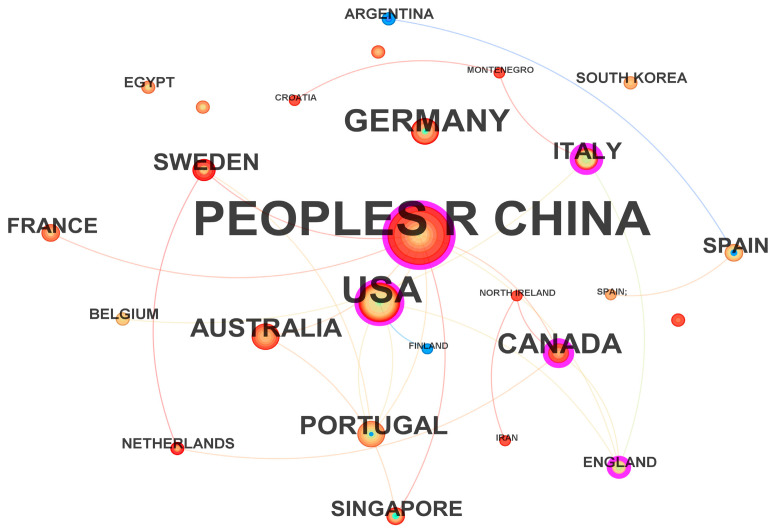
International collaboration network.

From the visualization of publishing institutions in Figure 5, it can be seen that institutions cluster around several major core organizations, with particularly close cooperation among Chinese institutions [51]. Circle size indicates that Beijing Institute of Technology and Tsinghua University have the highest publication outputs. As core institutions, they maintain extensive exchanges and collaborations with many organizations worldwide, reflecting strong influence in the global academic community. The results (Figure 6) show that China ranks first in publication volume, accounting for 51.9% of the total. This is largely attributable to China’s large automobile market and strong governmental support for new energy vehicles. Meanwhile, rapid advances in artificial intelligence have fostered a substantial research community and active related studies in China, highlighting its research capacity and influence in this field. The United States ranks second with 14.6%, followed by Germany (6.1%) and Canada (4.8%). The top five countries by citation frequency are China (2610), the United States (961), Norway (539), India (484), and the United Kingdom (395). The international collaboration network (Figure 7) shows that China and the United States maintain strong collaborative links with many countries, especially with each other, Australia, and Canada. Overall, both countries lead in publication output and citation frequency across nations and institutions.

To further enrich the interpretation of the international collaboration network (Figure 7), we discuss the complementarity of research orientations between leading institutions in China and the United States. Our keyword hotspot and evolution analyses indicate that the field has long been dominated by optimization- and control-oriented themes (e.g., MPC, vehicle dynamics, and optimization-based trajectory planning/tracking), while learning-based approaches (e.g., deep learning (DL) and RL) and system-level topics such as connected vehicles have gained increasing prominence in recent years. In this context, Chinese core institutions (e.g., Tsinghua University and Beijing Institute of Technology), which contribute a large share of publications and citations, are closely aligned with the optimization/control-driven mainstream that emphasizes constraint handling, stability, and engineering deployability. In contrast, U.S. core institutions (e.g., Virginia Polytechnic Institute and State University and the University of Texas at Austin) are more strongly positioned within the shift toward learning-based decision-making, interaction-aware planning, and connected/autonomous vehicle systems. This thematic complementarity offers a plausible explanation for the strong China–U.S. linkage observed in Figure 7: cross-national collaboration can integrate strengths in model-based optimization and control with advances in learning-based decision-making and system integration, thereby accelerating progress toward safer and more intelligent autonomous driving systems.

### 3.4. The Most Influential Publishing Institutions

According to Table 6, publisher-level statistics offer a complementary macro-level view of how autonomous driving research is disseminated and where influence is concentrated in this bibliometric dataset. In terms of publication volume, IEEE (159 papers), Elsevier (52), and MDPI (39) are the three most productive publishers, together contributing 250 papers. Within the top 10 list, IEEE alone accounts for 159 of 308 publications, highlighting its leading role as a major dissemination channel in this field.

A similar concentration is observed for citation impact. IEEE, Elsevier, and Springer Nature receive the highest citation counts (3129, 1768, and 289, respectively), totaling 5186 citations—approximately 89% of the citations accumulated by the top 10 publishers (5186/5835). Notably, publication volume and citation impact are not strictly proportional across publishers: citation intensity (citations per paper) varies, with Elsevier (≈34), Springer Nature (≈22), and IEEE (≈20) showing higher average citation rates in this dataset, whereas MDPI exhibits a lower average citation rate (≈4.7). Overall, these patterns suggest that a small number of major publishing systems host a large share of the literature and capture a disproportionate share of citations, thereby complementing venue- and author-level analyses in characterizing the field’s dissemination structure and impact distribution [52].

### 3.5. Research Field Analysis

**Figure 8:**
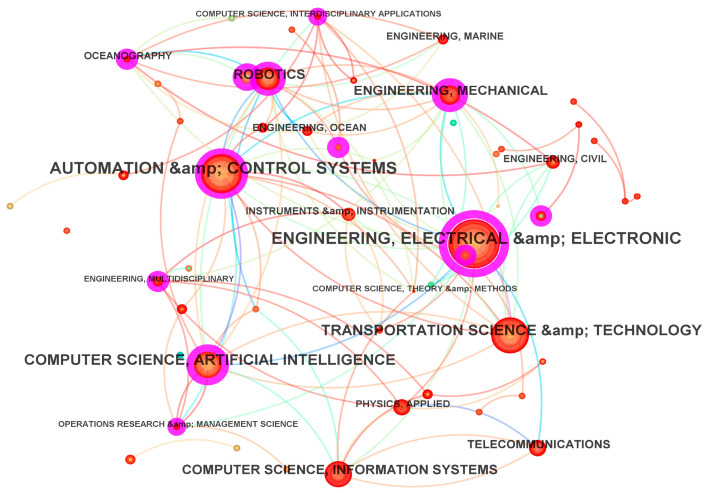
Main research areas and their relationships for autonomous vehicle path planning and trajectory tracking.

Research field analysis in CiteSpace is conducted by classifying the literature according to attributes such as WoS categories and research directions [53]. The research-field knowledge graph (Figure 8, Table 7) enables the identification of publication frequency and overlap relationships among 30 fields. Among them, engineering, electrical and electronics ranks first in both publication frequency and betweenness centrality, mainly because autonomous vehicle path planning and trajectory tracking span broad engineering topics, including real-time adaptability [54]. Automated Control Systems ranks second in publication frequency, but shows relatively low betweenness centrality, suggesting limited linkage with other fields. In contrast, computer science, oceanography, and robotics exhibit higher betweenness centrality, indicating stronger connections with other research areas.

In oceanography, research on autonomous underwater vehicles (AUVs) began earlier than that on ground AVs. Automated guided vehicles (AGVs) support a wide range of applications, spanning key topics such as optimal control, trajectory planning, and collision avoidance. In the development of ground AVs, certain algorithmic designs can draw on AUV-related studies [55], and the two research lines are highly correlated. Therefore, it is reasonable that Oceanography ranks second in betweenness centrality in this domain (0.38). Other fields in the top 10 include transportation science, telecommunications, and instruments and instrumentation.

### 3.6. Keyword Co-Occurrence Analysis

Keywords concisely represent the core concepts of a paper. In-depth keyword analysis effectively reveals the main thesis context. There must be a specific internal connection between the keywords listed in a paper, and this keyword co-occurrence frequency serves as a metric for the connection. There is a positive relationship between the co-occurrence frequency of keyword pairs and the strength of their thematic correlation [56].

**Figure 9:**
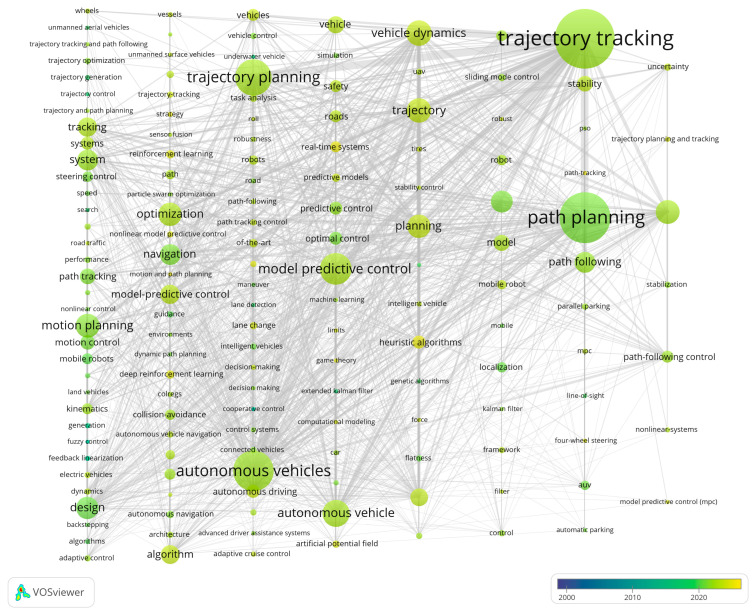
Keyword co-occurrence graph.

By analyzing the co-occurrence of word pairs, co-word analysis maps the conceptual structure and inherent linkages among topics in a document corpus. Specifically, by counting the frequency of each pair of subject terms in a set of documents appearing in the same document, a co-word network based on word pair associations can be constructed [57]. In this network, the number of nodes corresponds to the number of keywords in the graph, and the number of edges represents the number of connections between keywords. Whenever two keywords appear in the same document, a connection is formed between them. The keyword clustering in this field over the past 20 years is illustrated in Figure 9. In the visualization, each vertical column represents a keyword cluster. The color shift from blue to yellow denotes the average year of keyword emergence, a feature that serves to track the evolution of research hotspots within individual clusters.

The four-stage thematic evolution (2000–2007, 2008–2014, 2015–2019, and 2020–2025) was defined based on convergent bibliometric evidence rather than subjective partitioning. Specifically, the boundaries were determined by jointly considering: (1) the keyword time-overlay/average-year patterns from the co-occurrence clustering, (2) the temporal windows of burst keywords, and (3) structural changes in the publication-growth trajectory. Together, these indicators reveal major shifts in dominant themes and motivate the stage-wise interpretation.

From 2000 to 2007, autonomous vehicle planning and control technology was in its infancy, with relatively few related research results and literature. The keywords appearing in its key co-occurrence graph were also limited, mainly including feedback linearization, cooperative control, fuzzy control, and underwater vehicle. At this stage, research mainly relied on established control theories to support vehicle autonomous control. Given the strong nonlinear characteristics of complex control processes, it was necessary to simplify controller design and analysis and ensure stability near the equilibrium point; thus, feedback linearization methods were applied. In the co-occurrence graph of this period, feedback linearization is the largest node, followed by cooperative control, and the two main paths extending from “feedback linearization” are optimal control and AVs, indicating that optimal control in AVs was gradually gaining attention.

From 2008 to 2014, research in this field was in a slow development stage, and high-frequency keywords included design, navigation, localization, intelligent vehicles, trajectory generation, trajectory control, guidance, and cooperative control. Research gradually became more targeted and closer to practical applications. From the appearance design of AVs to vehicle positioning and navigation, these topics became key focuses, and researchers began exploring effective ways to achieve autonomous driving based on core AV technologies [20]. In positioning and navigation, GPS/IMU fusion and SLAM were widely explored to improve real-time accuracy and enable mapping/localization in unknown environments [58]. High-precision maps also supported more accurate path planning and traffic updates, improving system safety and reliability.

From 2015 to 2019, research continued to become more implementation-oriented, with trajectory generation and trajectory tracking control gaining prominence. In trajectory generation, methods such as Bezier curves were commonly used to smoothly connect waypoints and produce trajectories that satisfy vehicle dynamics constraints while considering comfort and feasibility. In trajectory tracking control, composite controllers combining feedforward and feedback integrated predictive capability with error correction, improving tracking accuracy and stability.

From 2020 to 2025, the field entered a period of rapid development, and high-frequency keywords included path planning, trajectory planning, motion planning, trajectory tracking, MPC, vehicle dynamics, and optimization. Path planning, vehicle dynamics modeling, and optimization algorithms became major hotspots, and researchers explored ways to enhance intelligent decision-making and control from the perspective of key AV technologies [21]. DL and RL were increasingly used for intelligent path planning in complex environments, while physics-based dynamics modeling combined with vehicle geometry and parameters supported accurate state prediction under various conditions [38]. Advanced optimization methods such as MPC, optimization-based trajectory planning, and multi-objective optimization were widely applied [22,59]. MPC predicts and optimizes future states through dynamic models to achieve accurate trajectory tracking; trajectory planning methods introduce constraints such as speed and comfort to generate optimal trajectories that conform to dynamics [23]; multi-objective optimization balances energy consumption, safety, and comfort to achieve optimal overall performance.

**Figure 10:**
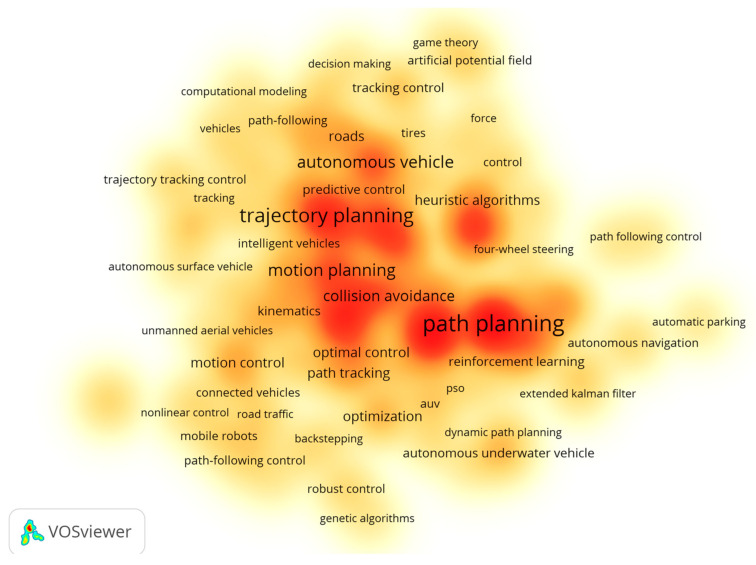
Keyword co-occurrence density map.

Highly cited papers often attract attention due to their significant breakthroughs and innovations in theory or technology, and their citation counts remain high. Research hotspots refer to specific topics that have received widespread attention and concentrated research in the academic community during a specific period of time [60]. In order to clearly observe the research hotspots of path planning and trajectory tracking in the field of autonomous driving, this study conducted a preliminary analysis of relevant data using CiteSpace software [19], and further used VOSviewer software to draw a keyword co-occurrence density map based on 329 high-quality and valid documents screened out (as shown in Figure 10). In this study, in order to accurately capture research hotspots, the minimum frequency of keyword appearance was set to 20 times. In the drawn graph, the gradual change of color from light yellow to dark red intuitively reflects the increasing frequency of keyword co-occurrence, that is, the increase in research enthusiasm. Through in-depth analysis of graph data from 2000 to 2025, it can be found that the research hotspots of path planning and trajectory tracking in the field of autonomous driving mainly focus on key technical contents such as path planning, trajectory planning, motion planning, collision avoidance, optimization, motion control, and RL [61]. It also covers important research directions such as connected and autonomous vehicles (CAVs) [62].

### 3.7. Analysis of Emerging Research Frontiers at Different Stages

The keyword burst map quantitatively depicts the popularity and temporal dynamics of research hotspots: a higher burst strength indicates greater research attention. Keyword burst detection was performed in CiteSpace over the period from 2003 to the present; because the number of records before 2003 was limited, the analysis starts from 2003. The settings were: time slice = 1 year, node type = keywords, and, given the manageable corpus size, all records retrieved for each year were included. Based on these settings, the top 25 keywords ranked by burst strength were extracted. In the figure, the red bar indicates the time interval during which a keyword shows a significant burst, i.e., when it exerts the strongest influence [63]. To interpret the burst keywords reports the quantitative burst characteristics of each keyword, including the first occurrence year, burst strength, and the beginning and ending years of the burst period. The keywords are ordered by decreasing burst strength.

**Figure 11:**
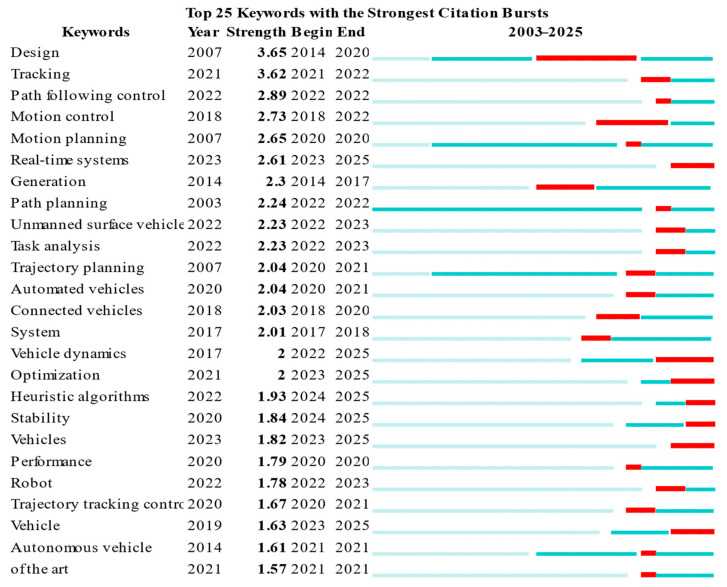
Keyword emergence map.

Early research hotspots included design, motion planning, trajectory planning, and path planning. Design emerged with the greatest intensity and duration, while motion planning, trajectory planning, and path planning, while first appearing earlier, emerged relatively later. This distribution characteristic indicates that the research focus in this field has gradually shifted from the early design stage that focused on basic theories and macro concepts to more practical stages such as implementation, optimization, and application. As shown in Figure 11, “motion control” emerged earlier than the other keywords and maintained a high level of popularity from 2018 to 2022, reaching a burst intensity of 2.73. This indicates that the performance requirements for motion control systems have continued to increase, and their applications have expanded to related fields such as AVs and robotics. The keywords tracing and path following control emerged in 2021 and 2022, with intensities of 3.62 and 2.89, ranking second and third, respectively, but the duration of emergence was less than one year. Since these two concepts are closely related to motion control, their research results and application scenarios overlap, but they focus more on vehicle trajectory tracking and therefore show higher emergence intensity in the short term.

The first appearance years of the vast majority of keywords are concentrated between 2018 and 2022, and their emergence duration is generally about one year, mostly concentrated within one to two years after the first appearance. In recent years, emerging research hotspots include real-time systems and heuristic algorithms. With the continued advancement of fields such as autonomous driving, the demand for real-time systems is becoming increasingly urgent, and the importance of reliable data processing capabilities has also increased significantly. At the same time, heuristic algorithms have also attracted widespread attention due to their efficiency and flexibility in handling complex optimization problems [21,64]. Another emerging hotspot focuses on vehicle dynamics and stability. As vehicle control systems continue to grow in size and complexity, stability analysis and control based on vehicle dynamics has become a key research direction.

To interpret the burst keywords in Figure 11 from a systems-engineering perspective, we map the emerging topics to the functional modules of an automated driving stack (Perception/Localization–Planning–Control) and their interfaces (Figure 1). Early bursts (e.g., design, motion/trajectory/path planning) mainly relate to the planning module, reflecting foundational formulations for route generation, maneuver feasibility, and constraint-aware trajectory synthesis. As research progressed toward implementation and deployment, the burst of motion control (2018–2022) indicates increased attention to the control module and its coupling with vehicle actuation, i.e., converting planned trajectories into low-level commands under drive-by-wire constraints (steering, braking, and propulsion). This shift also highlights the growing role of vehicle modeling and stability, since tracking performance depends on model fidelity and real-time compensation for dynamic effects. The short but intense bursts of tracing (2021) and path following control (2022) further emphasize the planning–control interface: beyond generating collision-free trajectories, the planned references must remain trackable under kinematic/dynamic limits and uncertainties, thereby motivating integrated or hierarchical frameworks that jointly consider trajectory generation and tracking control. More recently, the emergence of real-time systems and heuristic algorithms suggests increasing system-level constraints on computation and timing budgets, where planning and control must meet strict latency requirements while solving complex optimization problems. This has encouraged the use of fast heuristics and real-time-capable solvers on embedded platforms, and supports the broader adoption of predictive/optimization-based controllers in computationally efficient forms. Finally, the renewed focus on vehicle dynamics and stability highlights stronger cross-module coupling, where dynamics-aware modeling shapes not only controller design but also the planner’s constraint set (e.g., friction limits and stability margins), ultimately improving closed-loop safety and robustness.

Specifically, the burst keywords can be interpreted along the system information flow:

(1) Perception/Localization outputs (vehicle state, lane geometry, obstacle cues) enable planning feasibility.
(2) Planning outputs (reference path/trajectory) serve as inputs to tracking control.
(3) Control and actuation execute commands subject to dynamics and stability constraints.
(4) Real-time computation bounds the end-to-end latency of (1)–(3), motivating heuristics and efficient solvers.
(5) Vehicle dynamics/stability acts as a shared constraint layer that couples planning and control.

### 3.8. Most Influential Articles

Given that academic influence often takes time to accumulate and becomes apparent, and that the influence of an article will gradually expand and receive more citation feedback over time, this section uses citation frequency as a key indicator and comprehensively considers the impact of time factors on the influence of articles, thereby identifying the ten most influential papers between 2000 and 2023. First, as can be seen from Table 8, the article with the most citations is the article by Sahoo et al. [55], which reviews the research status and development trends of AUVs. The article focuses on the latest progress in positioning and navigation, path planning and control, sensor technology, and underwater communications. The article analyzes in detail various positioning and navigation methods, from inertial navigation to SLAM, and introduces different optimal path planning and control strategies. Fossen et al. [65] proposed a nonlinear adaptive path tracking controller to compensate for the vehicle side slip effect caused by drift forces from environmental factors such as ocean currents, wind, and waves. The method is based on the line of sight (LOS) principle and extends it to the tracking control of the Dubins path. The unknown sideslip angle is first modeled as a constant parameter, and by designing an adaptive law for online estimation, it is proved that the balance point between the lateral tracking error and the parameter estimation error is consistent semi-global exponential stability, thereby ensuring that the sideslip angle estimate converges exponentially to the true value. The proposed adaptive control law is essentially an integral LOS controller, and its parameter adaptation mechanism provides an integral action. This article is also the most cited article.

Eskandarian et al. conducted a comprehensive review of the multi-layer perception–planning–control architecture and key technologies of CAVs [66]. CAV enhances environmental perception and coordination capabilities through on-board sensors and vehicle communication technology, significantly improving the robustness and reliability of autonomous driving systems in complex environments. The article provides a systematic review of key autonomous driving technologies, delving into perception-layer methodologies for multi-sensor fusion, positioning, and mapping, planning-layer algorithms for decision-making and trajectory generation, and control-layer strategies for trajectory tracking. It also focuses on analyzing emerging research directions such as collaborative perception, multi-vehicle collaborative decision-making, and control brought about by networking, and the challenges they face. Luo et al. [62] developed a cooperative automated lane change strategy utilizing vehicle-to-vehicle (V2V) communication to address the problem in current autonomous driving research that lane change processes are difficult to avoid collision risks caused by changes in the state of other vehicles. The core of this strategy consists of two parts: trajectory planning and tracking control. By using lane change time and distance as constraint optimization variables, a reference trajectory that meets safety, comfort, and efficiency requirements is generated and updated in real time. This strategy is robust across diverse driving scenarios such as routine, emergency, and returning to the original lane. The proposed sliding mode controller achieves robust and accurate tracking of the planned vehicle trajectory.

Shen et al. proposed an integrated path planning and tracking control method based on rolling horizon optimization (RHO) for AUVs [67]. Considering the limited effective sensing range of onboard sensors, this study uses a spline path template to model path planning as an RHO problem. This approach defines the planned path as the state trajectory of a virtual reference system, which possesses an identical kinematic and dynamic model to the AUV. By constructing an appropriate error dynamics system, the AUV tracking control problem is transformed into a stabilization problem of the error system. The implemented Nonlinear MPC (NMPC) law inherently ensures the stability of the closed-loop system.

You et al. [68] proposed an autonomous lane-changing system for lane-changing behavior in autonomous driving, aiming to improve driving safety and alleviate traffic accidents and congestion caused by improper lane changes. The core of the system focuses on two major issues: trajectory planning and tracking control. The system uses a polynomial method to abstract vehicle motion as a time function to complete trajectory planning and uses an infinite dynamic circle to map collision detection to the parameter space. A backstepping-based tracking controller is designed, and its global convergence is verified by combining the Lyapunov function.

Dixit et al. [23] systematically reviewed the trajectory planning and tracking control methods in autonomous driving overtaking systems, pointing out that most current methods are only applicable to low-speed scenarios due to the uncertainty of environmental perception. The study compared different trajectory planning strategies from the perspectives of real-time performance, computational efficiency, and practical feasibility, pointing out that high-speed overtaking requires key considerations of vehicle dynamics, environmental constraints, and accurate perception of obstacles. The study addresses trajectory tracking by analyzing the advantages and disadvantages of various control algorithms and found that, despite the performance gains offered by advanced control methods, their effectiveness mostly depends on highly structured conditions, and existing schemes often assume that environmental information is completely known, which is inconsistent with actual driving conditions.

A fuzzy-logic-based system was employed by Antonelli et al. [69] to solve the motion planning and tracking problem for an autonomous robot on an unknown path. The system simulates human driving behavior and achieves efficient path following under the premise of satisfying the vehicle’s kinematic constraints (limited linear velocity, angular velocity, and acceleration). The method uses the approximate geometric information of the curve ahead as the input of the fuzzy system and outputs the cruising speed required to ensure safe passage in real time.

To address the AGV docking challenge, Chai et al. introduced a novel framework that integrates real-time trajectory planning with tracking control [70]. In the motion planning phase, the study employed a recurrent neural network (RDNN) architecture to perform DL approximations of the optimal parking trajectory, fully exploiting the inherent correlations between vehicle states. To enhance the planner’s adaptability, two transfer learning strategies were employed for deployment across various AGV platforms. To accurately track the planned trajectory, an adaptive learning neural network (ALNN) control algorithm was designed. Online adjustment of network parameters ensured both control system stability and tracking error convergence.

Zuo et al. [71] proposed a progressive MPC scheme (PMPCS) for intelligent vehicles. This scheme combines local path planning with tracking control and adopts an improved particle swarm optimization MPC method (IPSO-MPC) to solve both types of problems in a unified manner. This scheme significantly reduces the computational burden by seamlessly coordinating the two optimization layers. Furthermore, a novel planning algorithm is proposed that can handle both traffic light and overtaking timing constraints. The proposed algorithm incorporates MPC and artificial potential field (APF) to integrate time-varying safety constraints into the framework. These constraints are transformed into repulsive force fields and asymmetric lane potential fields to generate collision-free paths, while pseudo-speed planning is employed to achieve traffic scheduling under traffic light constraints.

Beyond citation counts, we further examine the interconnections among highly cited papers to reveal the knowledge structure underlying AV path planning and trajectory tracking. Using co-citation and bibliographic coupling links derived from WoSCC records, we find that the core literature forms several tightly connected clusters: (1) foundational review papers that are frequently co-cited as a conceptual baseline; (2) control- and optimization-oriented tracking studies (e.g., MPC-based controllers and controllers with stability guarantees); and (3) integrated frameworks that co-design planning and tracking. In addition, several highly cited papers play bridging roles by linking these clusters, suggesting their importance in transferring methods and problem formulations across subtopics. Overall, this relationship-based analysis complements citation-based ranking and provides a clearer picture of how core ideas and methods are organized and connected.

### 3.9. Co-Citations

**Figure 12:**
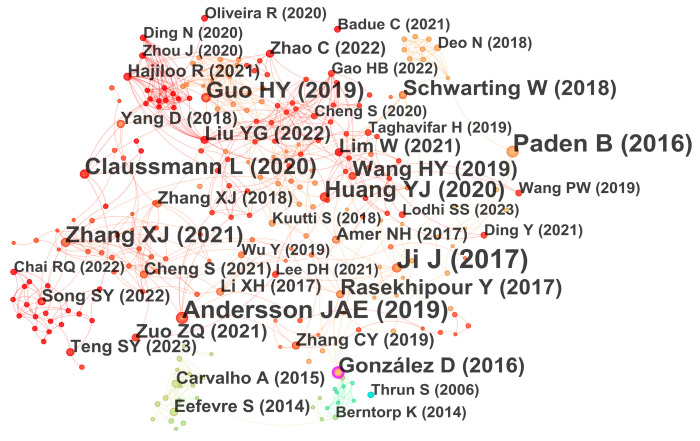
Co-citation map of literature.

The co-citation graph is an analytical technique applied to reveal the underlying relationship between documents and the structure of academic fields [72]. Its core is to identify the phenomenon that two or more documents are cited by a third subsequent document, thereby helping researchers to intuitively identify key documents, research hotspots, and the internal connections and development context between different directions in the field. In the co-citation graph presented in Figure 12, the dimension of the node corresponds to the citation frequency of the document: the larger the node, the more times the document has been cited and the more significant its influence. The color of the nodes changes over time, and the tones change from cold to warm, which intuitively presents the order of publication years: colder colors represent older documents, and the more reddish the color, the more recent the publication time. The arrows between nodes denote the co-citation relationship between documents. The existence of lines indicates that these documents are often cited by subsequent studies, thus reflecting their relevance in content or subject matter [19,73].

Analysis of Figure 12 shows that the node corresponding to Ji J (2017) is the largest, indicating the highest citation frequency (11 times). Its betweenness centrality is 0.09, ranking first among all nodes. The authors proposed an integrated framework for autonomous vehicle path planning and tracking, which generates collision-avoidance trajectories by constructing a three-dimensional virtual hazard APF and employs multi-constraint MPC for path tracking [74]. This approach achieves effective collision avoidance across multiple scenarios with strong dynamic performance and control stability, giving it high academic value and influence. Next are Paden B (2016) and Andersson JAE (2019), each with 9 citations. Paden B (2016) has a betweenness centrality of 0 and limited co-citation links, suggesting a weaker bridging role, likely because it is more often cited for review context and research objectives rather than as a direct theoretical basis. By contrast, Andersson JAE (2019) has a betweenness centrality of 0.07 and numerous co-citation links, indicating a stronger structural influence. The open-source numerical optimization framework CasADi, introduced by Andersson JAE, provides essential tool support for modeling and optimization in related studies and has therefore attracted broad attention and citations [75].

In addition, nodes such as Guo HY (2019), Zhang XJ (2021), Rasekhipour Y (2017) and Schwarting W (2018) also performed outstandingly in both citation frequency and betweenness centrality indicators, indicating that these documents are not only widely cited, but also play a key intermediary role in knowledge flow, confirming their core academic influence in this research field.

### 3.10. Overall Summary

This section provides an overall synthesis of the results presented in Section 3.1, Section 3.2, Section 3.3, Section 3.4, Section 3.5, Section 3.6, Section 3.7, Section 3.8 and Section 3.9. In general, the field has shown a sustained increase in research output, accompanied by a steady growth in academic impact, with a notable concentration across authors, institutions, countries, and publication venues. The thematic focus has gradually shifted from conventional control methods to application-oriented navigation/localization and trajectory-generation control. More recently, optimization-based approaches—particularly MPC—have progressed in parallel with data-driven methods represented by DL and RL. Keyword burst detection and the co-citation network further indicate emerging frontier shifts toward real-time implementation, heuristic algorithms, and vehicle dynamics and stability, and highlight important knowledge hubs such as integrated avoidance–tracking frameworks and optimization toolchains. The results are summarized in Table 9, which facilitates rapid identification of established evidence, research hotspots, and emerging trends, thereby supporting researchers in selecting appropriate directions based on their methodologies and data availability.

## 4. Discussion

### 4.1. Research Hotspots in the Field

Path planning and trajectory tracking of AVs have been a hot area of global research in recent years, and their development has lasted for decades. Although relevant research has been carried out since the 20th century, it was not until the early 2000s that substantial results and effective progress were achieved, driven by the autonomous driving competition held by the U.S. Defense Advanced Research Projects Agency (DARPA). From the initial attempts to use various sensors and verify the feasibility of different algorithms, to the subsequent simulation of driving in an urban environment, strictly abiding by traffic rules and actively avoiding other vehicles, this marks the transition of autonomous driving technology from the relatively simple wild environment to the complex and rule-driven urban environment [20]. As more and more car companies and technology companies enter the market, fully AVs are steadily moving towards the goal of commercialization.

**Figure 13:**
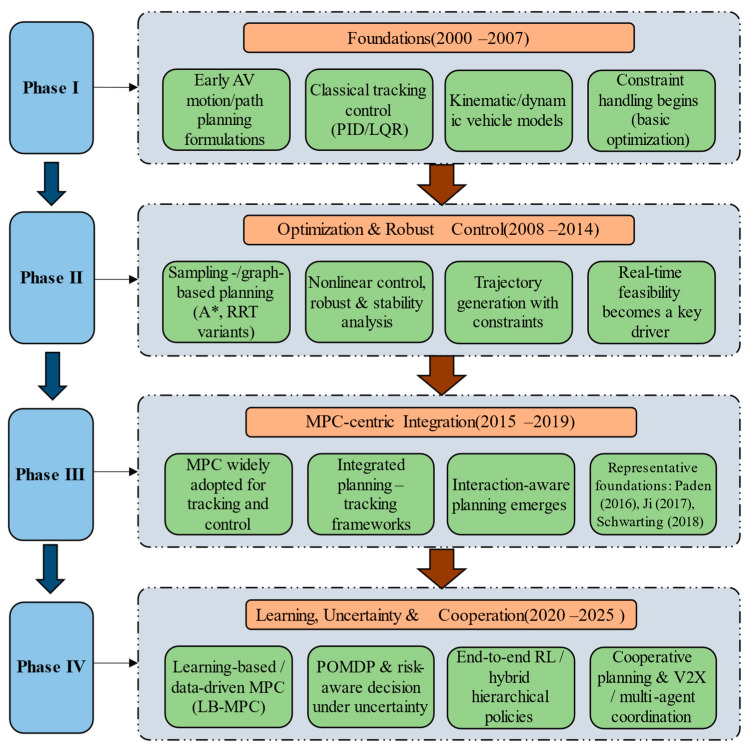
Thematic evolution and methodological roadmap of AV path planning and trajectory tracking (2000–2025).

So, given the current rapid development of autonomous vehicle path planning and trajectory tracking, what will be its future direction? Based on the bibliometric analysis presented in Section 3, we integrate research domain directions with core keywords and relate them to the thematic evolution and the methodological roadmap (Figure 13). From the perspective of current research hotspots, we discuss the main topics and summarize representative methods and key challenges.

The topic categories discussed in this section and summarized in Figure 13 were obtained by triangulating multiple outputs, including keyword clustering/density maps, burst detection, and highly cited/co-citation structures. We then mapped these data-driven clusters onto the autonomous-driving planning–control chain to provide a domain-consistent interpretation of how methodological paradigms evolve and how planning and tracking studies are positioned within the AV stack.

#### 4.1.1. Research on the Application of Path Planning and Trajectory Tracking Based on Vehicle Model

The vehicle model is a mathematical abstraction of the vehicle’s motion characteristics. It appears frequently in the keyword analysis above and can generally be divided into kinematic and dynamic models. The essence of model selection is to make a comprehensive trade-off between accuracy, real-time performance, and control performance. Ji et al. [76] clearly pointed out that whether it is path planning for lane changes or trajectory tracking using MPC, an accurate model is needed to predict the vehicle’s motion state in the future. Deng et al. [77] explored how to select a suitable vehicle model as a prediction model in MPC. The authors pointed out that it is crucial to strike a balance between the accuracy of the model and the computational complexity when selecting a model. They also mentioned that although more complex models can provide more accurate results, the amount of computation will also be greater, which will reduce the response speed of the control system. Therefore, a two-wheel three-degree-of-freedom model was selected as the prediction model. This model has a small error when tracking the vehicle trajectory and can output an ideal curve. Barcelo et al. [78] specifically discussed the issue of degree of freedom selection in vehicle modeling, pointing out that although more complex models can provide more accurate results, they require solving more ordinary differential equations, resulting in higher computational costs. The authors compared vehicle models with different degrees of freedom, including a 3-degree-of-freedom (DOF) bicycle model and a more complex 14-DOF full-vehicle model, pointing out that a 3-DOF model is sufficient in the conceptual design stage. Although building a more complex model is not an immediate need at present, it is crucial for comprehensively improving the ride comfort and personalized driving performance of AVs in the future.

Leveraging the principle of MPC, Qiao et al. [79] used kinematic and dynamic models as prediction models, designed two trajectory tracking controllers, and conducted simulation experiments. The results showed that both controllers had good dynamic tracking performance. The dynamic model demonstrated slightly superior performance compared to the kinematic model in both following effect and control smoothness, but under the same working conditions, its operation time was longer. Zhou et al. [80] investigated the trajectory tracking problem for a four-wheel independent steering (4WIS) vehicle under extreme conditions on intermittent ice-snow roads. Their study utilized a composite control method integrating sliding mode control (SMC) and tube MPC (Tube-MPC), based on a lateral 2-DOF dynamic model. The results showed that this method achieved a reduction of 0.54 m in the maximum lateral tracking error and the heading angle deviation by about 2.4 degrees under low adhesion roads (μ = 0.4). This scenario is characterized by an extremely low road adhesion coefficient, coupled with significant time-varying tire characteristics. The traditional kinematic model has obvious limitations in such scenarios [38]. This clearly demonstrates the necessity of high-order control algorithms based on dynamic models in low-adhesion and complex working conditions. In the selection of vehicle kinematic and dynamic models, basic scenarios can usually be effectively described by kinematic models; however, when faced with complex driving conditions, dynamic models often exhibit higher model applicability and control robustness due to their in-depth characterization of system coupling characteristics and mechanical responses [81]. Future developments in this field will focus on lightweight and intelligent models [82] and deeply integrate machine learning methods to learn dynamic features and behavior patterns from system operations in a data-driven manner, rather than relying entirely on traditional, precise physical modeling based on first principles.

#### 4.1.2. Research on Data-Driven MPC for Path Planning and Trajectory Tracking

In the keyword co-occurrence analysis, MPC also emerged as a core research hotspot in this field, showing a significant outbreak trend, especially between 2017 and 2023. This algorithm effectively models the driver’s underlying path-tracking behavior and decision-making logic. The framework uses the current vehicle motion state and a dynamic model to forecast the future motion trajectory and critical dynamic states over a finite horizon. By solving the online solution of a finite-horizon optimal control problem, the control input of the vehicle is calculated in real time, thereby achieving high-precision path tracking control [83]. With its inherent multi-constraint processing capability, the algorithm can effectively integrate road geometry parameters and vehicle kinematic constraints into trajectory tracking control, thereby ensuring robust feasibility and enhanced operational safety [81].

Based on the traditional MPC framework, more scholars continue to promote the performance optimization of algorithm content details. Sun et al. [84] developed an improved MPC approach method for achieving safe and efficient lane change operations. This method constructs a simplified vehicle model based on vehicle state and environmental information and uses a sigmoid function to constrain vehicle motion. A finite state machine (FSM) is used to select appropriate operations based on real-time driving conditions, and a discrete simplified dual neural network (SDNN) is introduced to quickly solve the quadratic programming problem to efficiently obtain the longitudinal and lateral accelerations required for lane change, braking, and overtaking. In practical applications, the significant nonlinear characteristics of vehicle models make it difficult for traditional modeling technologies to simultaneously achieve a balance between computational accuracy and real-time performance. To address this challenge, researchers such as Hobbani [85] proposed an innovative real-time feasible modeling and parameter identification method that employs a combined approach using particle swarm optimization (PSO) and the information criterion. By using the PSO algorithm to efficiently identify vehicle dynamic parameters and combining the information criterion to optimize the model structure, this approach notably reduces the computational burden while ensuring model accuracy, thereby providing a reliable and efficient model foundation for real-time vehicle control.

Currently, learning-based MPC (LB-MPC) has become a hot research topic in the field of MPC. This approach uses a well-trained data-driven model as a predictive model, integrating the excellent multi-constraint handling capabilities of MPC. Leveraging a more accurate predictive model, LB-MPC demonstrates enhanced robustness, particularly under extreme vehicle dynamics conditions. Han et al. [86] developed a learning-based model predictive path tracking control strategy. They constructed a 2-DOF single-track dynamic model of the vehicle, analyzed the single-step response error law of this model and the IPG Truck Maker model, and designed a method for constructing and rolling updating the error dataset. An error fitting model was established through Gaussian Process Regression (GPR), enabling real-time error compensation for the single-track model. Following this, the corrected model served as the prediction basis, with a dedicated cost function designed for path tracking, and a quadratic programming optimization problem was formulated, resulting in an LB-MPC framework for path tracking architecture. Xiao et al. [87] developed a modeling and control method for AVs that combines deep neural networks with the Koopman operator. This method uses DL to extend the dynamic mode decomposition algorithm, learns the finite-dimensional approximation of the Koopman operator, and designs a data-driven MPC controller based on this. This not only improves the generalization ability and interpretability of the controller but also makes it demonstrate superior performance in autonomous vehicle path tracking applications. High-fidelity CarSim simulations validate the effectiveness of the proposed method, showing that this method not only achieves high modeling accuracy over a wide operating range, but also has significant advantages in modeling performance over previous methods.

When facing more unknown and complex scenarios, we expect vehicles to have the ability to self-evolve in the implementation of specific functions. Yang et al. [88] achieved a novel integration of model-based and model-free RL, developing a method that leverages their respective advantages and proposed an integrated autonomous driving lane change strategy architecture based on a driving tendency network. They formulated the problem as an RL task and designed a comprehensive reward function to holistically address the decision-making and planning optimization. By applying the Temporal Difference MPC (TD-MPC) algorithm, an internal model was designed to predict future states and rewards, enabling local trajectory refinement within a short-term horizon. Parameter optimization of the driving tendency network was achieved through temporal difference learning based on long-term reward estimation. The method’s effectiveness was demonstrated through extensive testing in a high-fidelity simulation environment. The results showed that compared with traditional rule schemes, this method not only ensured driving efficiency but also significantly improved safety and comfort. In addition, compared with the soft actor-critic algorithm (SAC), this method achieved a 7 to 9 times improvement in learning efficiency.

At the same time, more meta-learning methods are being actively explored. For example, by enabling the Koopman model [87] to quickly adapt to new vehicles or environments, efficient learning can be achieved with minimal data, and the Koopman framework is applied to vehicle-to-everything (V2X) scenarios. These methods are expected to achieve safer and more coordinated planning and control behaviors.

#### 4.1.3. Research on the Application of Decision-Making Methods Based on Game Theory in Path Planning and Trajectory Tracking

Traditional path planning algorithms are generally predicated on the assumption of a static environment or considering the trajectories of other intelligent agents as known and fixed. However, in highly dynamic and highly interactive scenarios (such as autonomous driving on urban roads), such assumptions are difficult to hold. In reality, the decisions of intelligent agents are coupled to each other, and each party is often unable to accurately know the other party’s specific goals (such as aggressiveness or conservatism) and decision-making model [89]. Such interactions contain both conflicting goals (such as competition for spatial resources) and common interests (such as avoiding collisions), showing typical game characteristics.

Game theory offers a powerful mathematical foundation for modeling such interactive decision-making processes among multiple intelligent agents [90]. This framework can solve game solutions such as the Nash equilibrium and formulate the optimal response or equilibrium strategy for our side while fully considering the possible reactions of the other side. Applying game theory to path planning and trajectory tracking means that other intelligent agents (vehicles, pedestrians, etc.) are no longer simply viewed as obstacles, but as game participants with autonomous decision-making capabilities, their own goals, and the ability to respond rationally to our actions [91]. This method is particularly suitable for intense interaction scenarios such as merging and intersections. By predicting and optimizing the interaction results through mathematical models, it significantly improves the decision-making intelligence and drive safety of AVs in dynamic environments.

Zhang et al. [92] proposed a Stackelberg differential game-based MPC (DGTMPC) framework to address complex interaction and control problems in highway autonomous driving. The framework adopts a hierarchical structure: in the upper layer, vehicle interactions are modeled as a Stackelberg differential game, where the leader makes decisions first, and the follower responds accordingly, with both vehicles utilizing MPC for motion planning. The differential game is framed as a bilevel optimization problem, representing the objectives of the leader and the follower, and solved via a branch-and-bound algorithm. In the lower layer, a hybrid MPC is employed to simultaneously manage longitudinal dynamics (speed and acceleration) and discrete lane-level decisions (e.g., lane keeping or lane changing).

Furthermore, to improve the ability to identify the behavioral intentions of surrounding vehicles, this method also introduces an inverse MPC algorithm to estimate the target vehicle’s behavioral pattern. Experimental results show the significant effectiveness and superiority of the proposed framework in interactive lane-changing scenarios. Zhang et al. [93] proposed a Stackelberg game-based motion planning framework for autonomous driving to optimize vehicle behavior in dynamic interactive scenarios. The framework utilizes a hybrid path planner to model coupling relationships among traffic participants. Furthermore, it integrates a Stackelberg game-theoretic velocity planner with a quantitative leader-follower model, enabling AVs to accurately anticipate other agents’ responses and adapt their behavior accordingly. Through velocity planning optimization under corresponding strategies, the framework enhances autonomous vehicle control performance in interactive scenarios.

In the early prototyping of AVs, the initial approach was to ensure strict adherence to predefined traffic rules. However, it soon became evident that this was insufficient, as vehicles also needed to comply with certain unwritten human driving rules to integrate more naturally into the traffic environment. To address these challenges, Zanardi et al. [94] introduced a game-theoretic model termed Posetal Games. This model represents each participant’s preferences over outcomes using a partially ordered set, thereby integrating agents’ hierarchical priorities with environmental interaction characteristics. It is designed to tackle decision-making problems in multi-agent systems and has demonstrated particular effectiveness in trajectory selection for AVs. In urban autonomous driving scenarios, unwritten rules are more complex, and decision-making at unsignalized intersections is particularly challenging due to the lack of absolute regulations. To address this issue, Yi et al. developed a new framework for decision-making that leverages the concepts of intention prediction and mixed-strategy Nash equilibrium [95]. First, a combination of Gaussian Mixture Model–Hidden Markov Model (GMM-HMM) and SVM algorithms is used to predict the target vehicle’s driving intention at intersections (proceed to the left, to the right, or continue along the current path). Next, a trajectory fitting module leverages Bezier curves to generate the target vehicle’s predicted trajectory, based on the predicted intentions and road structure. By comparing this trajectory with that of the ego vehicle through an *s-t* diagram, potential spatiotemporal conflict points are identified. If conflicts exist, the mixed-strategy Nash equilibrium approach is applied to select the ego vehicle’s driving mode (yield or proceed). This method effectively avoids unnecessary early deceleration due to overly conservative behavior while preventing collisions or abrupt braking from overly aggressive maneuvers. Finally, based on the selected driving mode, the planning module leverages an MPC algorithm to derive the optimal acceleration strategy. Vehicle tests demonstrate that this decision-making framework ensures the ego vehicle can safely and comfortably navigate intersections. Therefore, game-theoretic decision frameworks can effectively balance safety, efficiency, and scalability while achieving reasonable decision-making in complex and dynamic driving behaviors.

#### 4.1.4. Research on the Application of Partially Observable Markov Decision Process (POMDP) in Path Planning and Trajectory Tracking

In real-world driving environments, the primary challenges for AVs arise from partial observability and uncertainty. For instance, due to limitations in sensing range and the coexistence of blind spots, vehicles may fail to detect pedestrians or other vehicles occluded by large vehicles or buildings, and the future behaviors of these traffic participants are inherently uncertain. POMDPs represent a principled approach to address such problems by maintaining a probabilistic belief over the intentions of other traffic participants and enabling optimal decision-making based on this belief.

To mitigate the aforementioned limitations, Kollarcik et al. [96] proposed a POMDP-based planning approach to handle uncertainty. Their methodology leverages the Adaptive Belief Tree (ABT) algorithm to address the POMDP problem approximately. The study first discretized the topology of intersections to facilitate the construction of the POMDP model. Additionally, a dynamic model was designed with the aim of predicting the evolution of vehicle states, including kinematic states like position and velocity, and an observation model was used to relate these states to actual observations, which may include noise. The results demonstrate that this approach can successfully plan collision-free trajectories across a series of simulation experiments, which utilize real-world traffic data from two different intersections. Jin et al. proposed a planning algorithm called BoT-Drive, which also addresses behavioral and trajectory-level uncertainties within the POMDP framework. Through driver models, BoT-Drive represents unknown behavioral intentions, enabling the inference of hidden driving styles from the model parameters. Framing the driver model as the AV’s decision-making behavior enables BoT-Drive to significantly alleviate the inherent exponential complexity of POMDPs. For improved safety and robustness, the planner employs importance sampling, utilizing high-level planned behaviors to refine driving trajectories. When benchmarked against state-of-the-art planning and learning-based methods on real-world data, BoT-Drive consistently surpasses them in diverse urban settings, from typical to complex, showcasing marked enhancements in driving safety and operational reliability [97].

To address the limitations of traditional behavioral decision-making methods in handling environmental uncertainty, which may lead to suboptimal decisions, existing studies have shown that approaches based on POMDPs demonstrate significant advantages over conventional reactive decision-making methods in terms of both traffic efficiency and safety [98].

#### 4.1.5. Research on the Application of Path Planning and Trajectory Tracking Based on End-to-End RL

In rule-based approaches, the environment is typically abstracted as a grid map or a sampling-based point set, and specific search algorithms are employed to compute a collision-free trajectory between the start and goal positions, often subject to certain optimality criteria. Subsequently, a controller enforces strict tracking of a preplanned spatiotemporal trajectory [99]. This approach offers strong interpretability and high execution efficiency, and it can guarantee optimal solutions in simple environments. However, when the environment changes dynamically, the entire graph structure must be updated, potentially triggering a re-search and significantly increasing computational load [100]. Furthermore, as the state-space dimensionality increases, search efficiency drops sharply, which can lead to computational delays or stalling and, consequently, pose safety risks.

In contrast, path planning driven by RL and trajectory tracking methods trains the vehicle in a variety of random scenarios to learn a strategy that directly outputs high-reward actions. These methods have the ability to adaptively adjust control behavior, effectively handle the complex nonlinear characteristics of the system, and demonstrate greater adaptability to dynamic environments. He et al. introduced a path planning and trajectory tracking algorithm built upon the Asynchronous Multi-threaded Proximal Policy Optimization (AMPPO) method [101]. By leveraging AMPPO, the computationally expensive online decision-making process is converted into offline training, enabling vehicles to autonomously learn planning, tracking, and emergency avoidance behaviors. The method mitigates the sparse reward problem by refining rewards at each time step and employing reward shaping techniques, while a goal-distance heuristic reward function is introduced to enhance directional guidance during exploration. Nevertheless, RL approaches face several challenges: they require training over millions of simulated or real-world trials, which is costly [102]; they encounter sim-to-real transfer difficulties; and the design of reward functions must be carefully considered to ensure their validity and effectiveness.

Although rule-based methods have certain limitations, their stability still has irreplaceable advantages [103]. Therefore, traditional rule-based methods can be combined with RL to form a complementary fusion architecture. For example, RL can be used to adaptively adjust and optimize traditional controller parameters [104]. Wen et al. [105] proposed a hybrid control algorithm that combines Deep Deterministic Policy Gradient (DDPG) with MPC to enhance overall system performance. Another approach involves pretraining RL policies using demonstration data generated by rule-based methods, thereby accelerating convergence and improving initial performance. In related work [106], data produced by MPC was used to train deep neural networks via imitation learning, which was then integrated into the DDPG framework with a feedforward exploration mechanism, significantly improving both learning efficiency and policy generalization.

A hierarchical hybrid decision architecture can also be adopted [102]: the upper layer generates a coarse-grained collision-free reference path or a series of sub-target points based on rule logic; the lower layer relies on a policy network trained by RL to output specific control instructions (such as velocity and orientation angle). To further ensure the robustness and safety of the system, the analysis methods of Lyapunov stability theory and traditional control theory can be integrated into the RL framework, for example, by designing a reward function or constraint condition based on the Lyapunov function to provide stability guarantees for the learning strategy [107]. A team from Tongji University proposed an online evolutionary decision-making framework for autonomous driving [108], which integrates data-driven DRL with model-driven MPC to enable online learning and evolution of AVs during operation. The framework follows a “longitudinal-priority, lateral-secondary” human-like decision logic, enhancing the interpretability of decisions. In the CARLA simulation environment, the framework achieved an 87% consistency with human driving behavior.

Although significant progress has been made in applying RL to path planning and trajectory tracking [109], numerous challenges and opportunities for further development remain. With the continuous advancement of machine learning techniques, future research may focus on joint optimization across different hierarchical levels, the development of RL algorithms with stability and safety guarantees [107], and the construction of more efficient multi-agent communication and coordination architectures.

Synthesizing the analysis and discussion of the five research hotspots mentioned above, the current research and future progress of autonomous vehicle path planning and trajectory tracking is summarized below: (1) Vehicle models are divided into kinematic models and dynamic models. The choice of models requires a trade-off between accuracy, real-time performance, and control performance. Accurate models are the basis for effective control, and different models have their own advantages in different scenarios. In the future, vehicle models will integrate machine learning technology and learn dynamic features in a data-driven way to improve performance and achieve lightweight and intelligent development. (2) As a core research hotspot in this field, MPC has significantly improved its performance in complex scenarios through the integration of machine learning and meta-learning methods, achieving high-precision path tracking and safe and efficient driving capabilities under the continuous optimization of researchers. (3) Game theory is widely used in path planning and trajectory tracking. By modeling the interactive decision-making process of multiple agents and considering the autonomous decision-making capabilities and reactions of other agents, it can achieve more intelligent and safe dynamic environment decision-making, especially in complex interactive scenarios (such as merging and intersections). (4) POMDP is used to deal with environmental uncertainty and partial observability. It makes optimal decisions by maintaining a probability distribution belief about surrounding agents’ intent, which can improve the efficiency and safety of vehicles in complex environments. (5) Rule-based and RL-based path planning and trajectory tracking methods each have their own advantages and disadvantages. A hybrid architecture that combines the advantages of both can improve the adaptability and the safety challenge for AVs posed by dynamic environments. Future research will focus on multi-level joint optimization, the stability and security of RL algorithms, and the efficiency of multi-agent collaborative architectures. Based on the hotspot research contents, we construct a structured knowledge table (Table 10). Table 10 integrates the main themes discussed in this section by linking representative methods with bibliometric evidence (keyword co-occurrence/occurrence, influential articles, and co-citation mapping) and lists the representative core references for each theme.

This structured summary complements the narrative discussion by providing evidence-based links between methodological trends and bibliometric findings, thereby enhancing interpretability and reproducibility.

### 4.2. Key Technical Bottlenecks and Future Research Directions

#### 4.2.1. Vehicle-Model-Based Planning and Tracking

A fundamental bottleneck lies in the accuracy–real-time–control-performance trade-off when selecting kinematic or dynamic models for planning and control. More complex models (higher DOF, richer tire/road coupling) can improve prediction fidelity and tracking smoothness, yet they often incur higher computational cost and slower controller response, which directly conflicts with the stringent real-time requirements of autonomous driving in urban and safety-critical scenarios. This challenge is amplified under extreme or low-adhesion conditions, where simplified kinematic assumptions break down, and time-varying parameters (e.g., tire characteristics, friction) create substantial model mismatch. Targeted future work should therefore focus on lightweight yet high-fidelity modeling [82]: (1) adaptive model complexity (switching/parameter-varying models) to match operating regimes [81,84]; (2) online identification and uncertainty-aware modeling to handle time-varying dynamics [80]; and (3) hybrid “physics + learning residual” paradigms that retain interpretability while compensating for unmodeled dynamics, enabling high-performance tracking without sacrificing real-time feasibility.

#### 4.2.2. Data-Driven MPC and Learning-Based MPC (LB-MPC)

For MPC-centered approaches, the main bottleneck is computational tractability under strong nonlinearity and multi-constraint coupling. In practice, nonlinear vehicle dynamics and complex constraints make it difficult for classical modeling and optimization to simultaneously satisfy accuracy and real-time execution, motivating simplified models, tailored solvers, or approximation strategies. LB-MPC alleviates part of this by using learned predictive models and error compensation (e.g., GPR residual correction, Koopman-based learning) [86,87], but it introduces new bottlenecks: distribution shift (learned models fail in unseen conditions), reliability of online updates, and closed-loop safety/stability guarantees when the predictive model is data-driven rather than physically grounded. To make future directions more targeted, research should prioritize: (1) real-time optimization acceleration (warm-starting, explicit/approximate MPC, learning-to-optimize) [104]; (2) robust/adaptive LB-MPC that explicitly models uncertainty and manages dataset shift via online validation and safe fallback mechanisms; and (3) theory-guided learning integration (e.g., bounded-error residual learning, constraint tightening/tube-based ideas) to provide feasible, explainable, and verifiable performance improvements in safety-critical driving tasks [87].

#### 4.2.3. Game-Theoretic Interactive Planning and Tracking

Game-theoretic methods provide a principled framework for modeling multi-agent coupling in interactive driving scenarios (e.g., merging and intersections). A major bottleneck, however, is the gap between idealized interaction assumptions and real-world traffic behavior [94,95]. In practice, an agent rarely has access to others’ true goals, aggressiveness, preferences, or decision models; thus, the interaction is partially observed, and classical rationality or equilibrium assumptions may be violated. Moreover, equilibrium selection ambiguity (when multiple equilibria exist) can further destabilize opponent-response predictions, potentially leading to overly conservative behaviors (reduced efficiency) or overly aggressive actions (compromised safety). Another bottleneck is scalability: as the number of interacting agents increases, equilibrium computation and multi-step strategic reasoning suffer from combinatorial growth in the joint strategy space and become increasingly sensitive to modeling assumptions. Accordingly, future research should prioritize: (1) integrating game-theoretic planning with intention prediction and behavior learning to reduce uncertainty about other agents; (2) bounded-rationality and risk-sensitive formulations that better capture human driving variability [89]; and (3) scalable approximations (e.g., hierarchical or graphical/mean-field games, local interaction decomposition, and receding-horizon game solving) that preserve safety and efficiency while enabling real-time deployment in dense urban traffic [91].

#### 4.2.4. POMDP-Based Decision-Making Under Partial Observability

POMDPs explicitly address uncertainty and partial observability by maintaining a belief over hidden states (e.g., surrounding agents’ intentions) [98]. However, a major bottleneck is computational complexity, particularly for continuous-state and continuous-action autonomous driving [110]. In real traffic, occlusions, blind spots, sensor noise, and inherently stochastic behaviors are common; although belief updates are principled, belief-space planning scales poorly due to high-dimensional continuous beliefs and the branching of action–observation trajectories, resulting in substantial online computation that challenges real-time deployment in complex urban environments. A second bottleneck is the strong dependence on observation and transition models: limitations in perception (e.g., missed/false detections, occlusion uncertainty, and imperfect noise/time-delay characterization) can propagate to biased belief estimates and, consequently, suboptimal decisions [96]. Accordingly, promising future directions include: (1) hierarchical and approximate POMDP frameworks (e.g., discrete topological behavior planning with continuous control, or high-level intention/behavior planning coupled with low-level MPC) to reduce online burden; (2) learned or amortized belief representations that compress high-dimensional uncertainty while retaining safety-critical information; and (3) tighter perception–planning coupling via uncertainty-aware perception outputs and robust belief updates (e.g., risk-sensitive/chance-constrained planning), so that partial observability is quantified and managed explicitly rather than implicitly ignored.

#### 4.2.5. Rule-Based and End-to-End RL and Hybrid Architectures

End-to-end RL can shift costly online decision-making to offline training and enables the acquisition of complex behaviors [101]; however, it still faces several practical bottlenecks, including sample inefficiency and high training cost (often requiring a large number of interactions), sim-to-real transfer challenges, and sensitivity to reward design, where poorly specified rewards may lead to unsafe or unintended strategies. In safety-critical autonomous driving, an additional bottleneck is verifiability: purely learned policies are difficult to interpret and to certify in terms of stability, constraint satisfaction, and robustness [107]. As discussed in our manuscript, hybrid designs that combine rule-based or optimization-based safety envelopes with RL-based adaptability (e.g., RL for controller-parameter tuning, RL–MPC integration, hierarchical hybrid decision architectures, and Lyapunov- or barrier-informed constraints) offer a promising path forward. Accordingly, future work should prioritize: (1) safe and constrained RL with explicit stability/safety guarantees (e.g., barrier- or Lyapunov-guided learning); (2) hybrid, multi-level joint optimization that leverages rules/optimization for safety assurance while using RL for adaptation; and (3) data-efficient learning and robust generalization (e.g., imitation/offline RL with safety validation, domain randomization with closed-loop real-world refinement) [111], to enable practical deployment while maintaining safety, interpretability, and robustness in dynamic environments.

Overall, research in the domain of path planning and trajectory tracking for autonomous systems is moving toward multidisciplinary integration. By integrating kinematic and dynamic models, MPC, game theory, POMDPs, and rule-based and RL methods, combined with machine learning techniques and data-driven dynamic feature learning, lightweight and intelligent vehicle models can be constructed to improve decision-making intelligence and driving safety in complex dynamic environments. This will become a key development direction for future autonomous driving technology. Meanwhile, in response to the key bottlenecks exposed by the above hotspot technical routes—such as the trade-off between model accuracy and real-time performance; online optimization and provable safety in MPC/LB-MPC; intention uncertainty and scalability in interactive game-theoretic settings; belief-space complexity and perception-error propagation in POMDPs; and the sample efficiency, sim-to-real transfer, and limited verifiability of end-to-end RL—future research should pursue more targeted breakthroughs centered on the core objective of “robustness–real-time performance–interpretable/verifiable safety.” This can be achieved by synergistically combining physics-informed priors with data-driven learning, and by co-designing hierarchical/hybrid architectures with explicit safety-constraint mechanisms, thereby closing the loop from hotspot directions to bottleneck identification and actionable research pathways, and ultimately supporting reliable deployment in complex traffic environments.

## 5. Conclusions and Future Work

### 5.1. Conclusions

This article, using literature visualization software, created a knowledge graph and conducted a multi-dimensional visualization analysis of the literature in the field of autonomous driving path planning and trajectory tracking from 2000 to 2025, covering publication volume, influential authors, core institutions, major publishing institutions, keyword co-occurrence, and co-citations. By thoroughly analyzing keyword-related research hotspots, we comprehensively reviewed research progress in this field and reached the following conclusions:

(1) In terms of the number of publications and the time of publication, research in the field of autonomous vehicle path planning and trajectory tracking has been on the rise, especially in the past five years.
(2) In terms of main authors, Li Keqiang’s team at Tsinghua University ranks first in terms of the number of publications and citation frequency, with strong academic influence and wide recognition; other influential authors include Chen Yimin’s team and Bitar Glenn’s team.
(3) Tsinghua University, the Norwegian University of Science and Technology (NTNU), and the Beijing Institute of Technology (BIT) lead this field in both publication volume and citation impact. Their prolific output establishes them as the foremost research institutions.
(4) The main contributions in this field originate from a select group of countries, namely China, the United States, Norway, India, and the United Kingdom. Furthermore, China exhibits the most extensive international collaboration network, working closely with partners such as the United States, Australia, and Canada.
(5) In terms of publishing institution influence, IEEE, Elsevier, and MDPI are the main publishing platforms, accounting for 76% of the total publication volume. Among them, IEEE has a particularly significant influence due to its authoritative position in the field of electrical and electronic engineering.
(6) From the perspective of research field distribution, engineering, electrical and electronic engineering, automated control systems, and computer science are the main research directions in this field. The publication frequency and betweenness centrality values of these fields are relatively high, indicating that they occupy a core position in academic research.
(7) Keyword co-occurrence results show that trajectory tracking, trajectory planning, motion planning, and MPC appear most frequently, reflecting the core technologies in this field. A keyword emergence analysis identifies DL and RL as rising trends, highlighting their growing application in creating path planning and trajectory tracking solutions for AVs.
(8) Judging from the co-citation map of the literature, the literature nodes of Ji J (2017), Paden B (2016), and Andersson JAE (2019) are the largest, among which the literature of Ji J (2017) ranks first in both citation frequency and betweenness centrality, highlighting the important position of the author in this subject field.

### 5.2. Future Work

Research interest in the field of path planning and trajectory tracking for AVs continues to rise, with the number of publications reaching new historical peaks year by year. This not only reflects the rapid development of autonomous driving technology but also reflects the accelerated advancement of its marketization and commercialization process. However, only vehicles that reach Level 4 or higher are truly considered AVs. Within the system’s designed operational domain (ODD), these vehicles are capable of safely and autonomously completing driving tasks without human intervention. However, this also complicates the allocation of responsibility for accidents. Therefore, from a technical perspective, the robustness and safety of autonomous vehicle path planning and trajectory tracking still need to be further improved, especially when interacting with other traffic participants, so that they can make safe and efficient decisions. In the future, the development direction of this field will mainly focus on the following two aspects:

(1) In the future, as DL algorithms continue to evolve, end-to-end autonomous driving models will become the mainstream trend. These models integrate multiple modules, such as decision-making, planning, and control, into a unified neural network, directly mapping raw sensor inputs to vehicle control commands or driving trajectories. End-to-end learning avoids the information loss and error accumulation associated with traditional modular architectures, enabling joint optimization of all links and significantly improving the overall performance and generalization capabilities of the system. Large models based on the Transformer architecture, in particular, demonstrate significant potential for processing multimodal data, understanding complex scenarios, and long-term dependencies due to their powerful sequence modeling and parallel processing capabilities. As computing power increases and data volumes accumulate, autonomous driving models with larger parameters and stronger capabilities will continue to emerge. These models will combine advanced AI technologies such as RL, imitation learning, and world models to enable vehicles to approach or even surpass human driving capabilities in path planning and trajectory tracking, providing stronger support for the safe and efficient operation of AVs.
(2) As a pivotal element of intelligent transportation systems, V2X technology offers robust support for the navigation and motion control of highly AVs through improved path planning and trajectory tracking. Through V2X communication, vehicles can exchange information in real time with all relevant entities, encompassing surrounding vehicles (V2V), roadside infrastructure (V2I), pedestrians (V2P), and cloud networks (V2N/V2C), providing a broader perspective and earlier warnings. V2X technology can also help vehicles achieve collaborative driving, such as platooning, to improve traffic efficiency and reduce energy consumption. With the popularization of 5G/6G communication technology and the widespread deployment of roadside intelligent devices, V2X technology will be deeply integrated with single-vehicle intelligence to jointly build a safer, more efficient, and smarter future transportation system, making the path planning and trajectory tracking of AVs more accurate and reliable.

The content of this article still needs to be improved. First, the data source mainly relies on electronic databases. Even if the WoS is used in conjunction with the data, there may still be problems with the missed or incorrect selection of literature. Future research could consider expanding data sources, incorporating more databases, and utilizing more efficient screening tools to improve the accuracy of literature screening. Secondly, currently used bibliometric software still relies on the researcher’s judgment when clustering themes, which, to some extent, affects the objectivity of the results. Furthermore, the depth of current research on the application of keyword networks in bibliometric analysis still needs to be improved. With the continued advancement of DL and natural language processing technologies, future research in this field is expected to achieve a higher level of automation and precision. DL models can effectively identify key information in texts, extract it as keywords, and construct network structures based on this information, thereby more clearly revealing the inherent connections and knowledge context between documents.

## Figures and Tables

**Table 1:** Multi-dimensional comparison between representative review studies and the present study.

Aspect	Paden et al. [20]	Schwarting et al. [21]	Yu et al. [22]	Dixit et al. [23]	This Study
Scope	Motion planning + control for self-driving urban vehicles	Planning & decision-making for AVs	MPC for AVs	Overtaking scenario: trajectory planning & tracking	Path planning + trajectory tracking (2000–2025)
Evidence base	Selected/curated papers	Selected/curated papers	Selected/curated papers	Selected/curated papers	WoSCC; 329 papers after screening
Publication year	2016	2018	2021	2018	2025
Coverage window	Not specified	Not specified	Not specified	Not specified	WoSCC 2000–2025, PRISMA screened, n = 329
Methodology	Narrative survey/taxonomy	Narrative synthesis	Algorithm-focused review	Scenario-specific review	CiteSpace/VOSviewer + co-word/burst + topic evolution + LDA
Outputs	Conceptual taxonomy, challenges	Problem framing, methods overview	MPC variants, constraints, implementation issues	Comparison by real-time, feasibility, etc.	Trends, core authors/institutions, hotspots, frontiers, topic evolution

**Table 2:** Subject keywords.

Search Keywords
“Autonomous” OR “Self-Driving” OR “Driverless” OR “Self-Piloting”
“Motion Planning” OR “Trajectory Planning” OR “Path Planning”
“Trajectory Tracking” OR “Path Following”
“Vehicle” OR “Car” OR “Automobile” OR “Motor Vehicle” OR “Motorcar”

**Table 3:** Data regarding authors with the highest number of publications.

Author	Publications	Institution	Country
Li K.Q.	5	Tsinghua University	China
Chen Y.M.	4	Northwestern Polytech University	China
Melchior P.	3	Centre National de la Recherche Scientifique	France
Pascoal A.	3	University of Lisbon	Portugal
Nie Z.G.	3	Kunming University of Science & Technology	China
Di Cairano S.	3	Mitsubishi Electric Research Laboratories	USA
Yue M.	3	Dalian University of Technology	China
Lian Y.F.	3	Changchun University of Technology	China
Marzbani H.	3	RMIT University	Australia
Receveur J.-B.	3	University of Bordeaux	France

**Table 4:** Authors with the highest citation counts (top 10).

Author	Publications	Citations	Country
Sahoo A., Dwivedy S. K., & Robi P. S.	2	454	India
Li Keqiang	5	388	China
Shi Yang	2	268	Canada
Luo Yugong	2	251	China
Lie Guo	2	210	China
Shen Chao	2	205	Canada
Wang Junmin	3	174	USA
Luo Xiaoyuan	2	129	China
Chu Duanfeng	2	120	China
Zhao Chenyang	2	102	China

**Table 5:** Institutions with the highest citation counts (top 10).

Institution	Publications	Citations	Country
Tsinghua University	10	545	China
Norwegian University of Science Technology	6	538	Norway
Beijing Institute of Technology	17	504	China
National Institute of Technology System	3	490	India
Indian Institute of Technology System	7	484	India
Wuhan University of Technology	3	347	China
Chinese Academy of Sciences	3	275	China
Virginia Polytechnic Institute State University	3	241	USA
University of Texas Austin	4	223	USA
Dalian University of Technology	5	221	China

**Table 6:** Ranking of the top 10 publishing institutions in both publication volume and citation count.

Publishing Institutions	Publications	Citations
IEEE	159	3129
Elsevier	52	1768
MDPI	39	185
Sage	21	268
Springer Nature	13	289
Wiley	8	132
Taylor & Francis	6	40
Amer Soc Mechanical Engineers	4	2
Hindawi Publishing Group	3	17
Inderscience Enterprises Ltd.	3	5

**Table 7:** Analysis of the top 10 research fields by both frequency and betweenness centrality.

Research Field	Publications	Centrality
Engineering, electrical and electronic	201	0.49
Automation control systems	106	0.12
Computer science	103	0.26
Transportation science	64	0.09
Robotics	50	0.15
Telecommunications	26	0
Instruments instrumentation	19	0.01
Oceanography	17	0.38
Physics	15	0.07
Operations research management science	12	0.11

**Table 8:** Top 10 papers ranked by citation count.

Paper	Year	Times Cited
Advancements in the field of autonomous underwater vehicle [55]	2019	453
Line-of-sight path following for Dubins paths with adaptive sideslip compensation of drift forces [65]	2014	453
Research advances and challenges of autonomous and connected ground vehicles [66]	2019	239
A dynamic automated lane change maneuver based on vehicle-to-vehicle communication [62]	2015	225
Integrated path planning and tracking control of an AUV: a unified receding horizon optimization approach [67]	2014	204
Trajectory planning and tracking control for autonomous lane change maneuver based on the cooperative vehicle infrastructure system [68]	2015	196
Trajectory planning and tracking for autonomous overtaking: State-of-the-art and future prospects [23]	2018	176
A fuzzy-logic-based approach for mobile robot path tracking [69]	2016	164
DL-based trajectory planning and control for autonomous ground vehicle parking maneuver [70]	2022	158
MPC-based cooperative control strategy of path planning and trajectory tracking for intelligent vehicles [71]	2020	127

**Table 9:** Comparative matrix of key findings across Section 3.1, Section 3.2, Section 3.3, Section 3.4, Section 3.5, Section 3.6, Section 3.7, Section 3.8 and Section 3.9 (2000–2025).

Section	Key Finding	Key Representative Objects	Research Implications
3.1	The field exhibits a sustained growth trajectory in both publication output and citation impact, with an accelerated expansion after 2014 and a marked increase after 2020; the comparatively low citation count in 2025 is largely attributable to citation lag.	Peak annual output occurs around 2024 (~50 papers); citations increase sharply during 2021–2024.	These trends support the continued research vitality of path planning and trajectory tracking and provide a temporal rationale for further investigations.
3.2	According to Price’s Law, the core-author threshold is approximately two publications, yielding a sizeable core group; the collaboration structure is characterized by several clusters organized around a small number of hub authors.	Core authors: 94; highest productivity: 5 publications.	The identified hubs and clusters provide practical entry points for benchmarking, collaboration building, and systematic tracking of emerging contributions.
3.3	Research productivity and influence are highly concentrated at both institutional and national levels; China contributes the largest share of publications and citations, while China–USA links constitute a central axis in the international collaboration network.	China: 51.9% of publications; China citations: 2610; USA: 14.6% of publications; influential institutions include Tsinghua University and NTNU.	The institutional–national landscape informs strategic collaboration planning and helps situate new studies within the global research ecosystem.
3.4	Publication channels are strongly concentrated among major publishers (IEEE, Elsevier, and MDPI), while citation performance differs across publishers, indicating that publication volume and scholarly influence are not necessarily aligned.	Paper counts: IEEE 159; Elsevier 52; MDPI 39; citation leadership is dominated by IEEE/Elsevier/Springer Nature.	Venue selection should consider both dissemination capacity and expected citation impact, rather than publication volume alone.
3.5	The dominant Web of Science categories are electrical and electronic engineering, automation and control, and computer science; notably, oceanography shows relatively high betweenness centrality, suggesting cross-domain methodological transfer from AUV-related research.	Engineering, electrical and electronic: frequency 201, centrality 0.49; oceanography centrality 0.38.	The disciplinary distribution motivates interdisciplinary framing and encourages leveraging transferable methodologies from adjacent autonomy domains (e.g., AUV/robotics).
3.6	Keyword evolution indicates a three-stage thematic progression: early emphasis on classical control (2000–2005), subsequent focus on navigation/localization and trajectory generation (2006–2016), and a recent shift towards optimization-based approaches (e.g., MPC) with increasing attention to DL and RL (2017–2023).	Stage definition: 2000–2005/2006–2016/2017–2023.	This progression provides a structured basis for positioning new contributions and for articulating novelty relative to stage-specific research hotspots.
3.7	Burst detection highlights frontier transitions: motion control demonstrates a sustained burst over 2018–2022, whereas tracing and path following control show strong short-term bursts; additional emerging themes include real-time systems, heuristic algorithms, and vehicle dynamics/stability considerations.	Burst strength: tracing 3.62; path following control 2.89; motion control 2.73.	Burst signals may be used to prioritize near-term research opportunities and to justify topic selection based on attention shifts within the community.
3.8	Highly cited publications delineate the intellectual base of the field, with strong influence from AUV-related surveys/control foundations and representative directions including V2V cooperative lane change, integrated planning–tracking frameworks, deep-learning-based parking, and cooperative MPC.	Highest citation count in Top 10: 453.	The high-impact set provides a core reading backbone for literature reviews and facilitates mapping seminal methods onto specific application contexts.
3.9	Co-citation analysis identifies foundational hubs in both methodology and tooling: Ji J (2017) represents an influential integrated framework combining artificial potential fields with constrained MPC for collision-avoidance trajectory generation and tracking, while CasADi-related work constitutes a key optimization modeling reference.	Ji J (2017): co-cited 11 times; betweenness centrality 0.09.	Hub references support the selection of baseline frameworks and optimization toolchains and motivate methodological extensions for planning–control integration.

**Table 10:** Summary of major research themes, representative methods, bibliometric evidence, and key references.

Discussion Theme	Vehicle Model–Based Path Planning & Trajectory Tracking	Data-Driven MPC/Learning-Based MPC (LB-MPC)	Game Theory–Based Interactive Decision/Planning & Tracking	POMDP Under Uncertainty for Planning & Tracking	End-to-End RL for Planning & Tracking
Representative methods	Kinematic/dynamic vehicle modeling model-based planning + tracking; model-based MPC/receding horizon; stability-guaranteed controllers (e.g., Lyapunov-based tracking)	Data-driven identification for predictive models; LB-MPC integrating learning with MPC; Koopman-based modeling meta-learning assisted MPC; safety/robust constraints within MPC	Stackelberg/differential games; game-theoretic MPC; interaction-aware planning; intent inference for surrounding agents; equilibrium/strategy-based decision making	Belief-state planning; POMDP-based behavior planning under partial observability; probabilistic inference of hidden states/intent; approximate solvers; risk-aware decision-making	Deep RL/end-to-end policy learning; hierarchical hybrid decision architectures (high-level decision + low-level control); imitation + RL; multi-agent RL with coordination/communication
Typical scenarios & challenges	Works well in structured environments with relatively reliable dynamics; key challenges: modeling errors (model mismatch), parameter uncertainty, and real-time feasibility under constraints	Targets unknown/complex scenarios where accurate first-principles models are hard; challenges: stability & safety guarantees, robustness under distribution shift, sample efficiency, and real-time computation	High interaction scenarios (lane change, merge, overtaking); key challenges: multi-agent coupling, behavior uncertainty, computational burden of equilibrium solving, and ensuring safety/comfort under interactive constraints	Real-world uncertainty (occlusion, hidden intentions, incomplete perception); challenges: computational complexity, belief update cost, and integrating uncertainty-aware decision with low-level tracking/control	Complex and high-dimensional tasks; challenges: safety and constraint satisfaction, interpretability, sim-to-real transfer, training stability, and multi-agent coordination
Bibliometric evidence	(1) Vehicle models and dynamics are repeatedly emphasized in the keyword analysis (Section 3.6 and Section 3.7); (2) multiple highly cited works focus on integrated planning–tracking with model-based optimization (Section 3.8); (3) structural influence of foundational works is reflected in the co-citation mapping (Section 3.9)	(1) MPC is highlighted as a core hotspot in keyword co-occurrence analysis (Section 3.6) and discussion; (2) emerging-frontier discussion aligns with keyword emergence (Section 3.7); (3) influential works include learning/DL and cooperative MPC (Section 3.8)	(1) Interaction/multi-agent direction is consolidated in the stage-wise frontier analysis (Section 3.7); (2) connected/cooperative driving is reflected in influential works and network structures (Section 3.8 and Section 3.9)	(1) Identified as a key frontier theme in Discussion and consistent with emerging-frontier logic (Section 3.7); (2) can be linked to co-citation clusters related to uncertainty/decision-making (Section 3.9)	(1) Learning-based methods are emphasized as later-stage hotspots consistent with Section 3.7; (2) high-impact DL planning/control works appear in the influential paper list (Section 3.8)
Representative core references	[67,68,71,76,77,78,79,80,81,82]	[70,71,83,84,85,86,87,88]	[62,90,91,92,93]	[96,97,98]	[70,99,100,101,102,107,108,109]

## Data Availability

The data presented in this study are available on request from the corresponding author.
